# Effects of *Tremella fuciformis* Powder with Varying Particle Sizes on the Gel Properties of Soy Yogurt

**DOI:** 10.3390/foods15061000

**Published:** 2026-03-12

**Authors:** Songze Li, Ziying Fang, Xiaoping Yang, Jianfeng Wu, Xiang Fang

**Affiliations:** 1 College of Food Science, South China Agricultural University, Guangzhou 510630, China; lisongze0323@163.com (S.L.); 15828159606@163.com (Z.F.); yangxiaoping0929@126.com (X.Y.); 2 Danling Institute of Food Bio-Intelligent Manufacturing, South China Agricultural University, Meishan 620200, China

**Keywords:** *Tremella fuciformis* powder, particle size, soy yogurt, gel properties, filling effect

## Abstract

This study aimed to elucidate the mechanisms by which *Tremella fuciformis* powder (TFP) improves the gel properties of soy yogurt by investigating the effects of TFP particle size on physicochemical properties and rheological behavior, combined with microstructural characterization and intermolecular interaction analyses. The results demonstrated that reducing TFP particle size (from 432.33 µm to 50.10 µm) significantly enhanced its hydration properties and increased the water holding capacity of soy yogurt to 99.44% (*p* < 0.05). Rheological analysis showed that reduced TFP particle size increased the apparent viscosity, storage modulus, and loss modulus of soy yogurt, consistent with the formation of a denser gel network observed via particle size analysis and SEM. While larger particles disrupted ordered protein cross-linking, smaller TFP particles enhanced soy yogurt gel properties by filling voids, during which volumetric expansion through water absorption reinforced gel matrix continuity. Zeta potential and intermolecular interaction analyses suggested that reduced TFP particle size enhanced non-covalent interactions in soy yogurt. These results indicate that TFP improves the gel properties of soy yogurt primarily through filler effects and protein interactions, providing a valuable reference for formulating stable plant-based yogurt products.

## 1. Introduction

The challenges of increasing public awareness of food security and global population growth are driving innovation in healthy and sustainable protein alternatives [1,2]. Plant-based foods are recognized as an optimal protein alternative, owing to their wide availability, absence of ethical concerns, and lower environmental impact [3]. Plant-based yogurt represents a significant segment of non-dairy alternatives. Through microbial fermentation, it achieves a texture and mouthfeel comparable to traditional dairy yogurt [4]. Among plant-based yogurt products, soy is the most extensively utilized base material. Soymilk has been demonstrated to be an excellent culture medium for the growth of lactic acid bacteria [5,6]. However, the weak gel structure formed during the acidification process of soy yogurt often leads to phase separation [7], compromising consumer acceptance. Incorporating thickeners to stabilize colloidal systems and facilitate structural organization is an important strategy for enhancing the gel stability and sensory quality of soy yogurt [8], such as carboxymethyl cellulose, resistant starch, and gellan gum [9,10,11]. These thickeners enhance the structural organization of the protein network, increase water binding capacity, elevate system viscosity, and modulate polysaccharide–protein interactions, thereby improving the overall system stability [12]. Nevertheless, a conflict persists between thickener use and clean-label trends [13]. Consequently, recent research has shifted towards natural bio-based alternatives that enhance technical functionality while delivering health benefits [14,15]. Studies have demonstrated that the functional properties of naturally derived hydrogels largely depend on their molecular structural characteristics, particle properties, and interaction behaviors with protein matrices [16,17,18]. Therefore, natural ingredients that combine strong water holding capacity with high compatibility with protein matrices have emerged as a research hotspot in the field of food colloids and are considered promising alternatives to conventional thickeners.

*Tremella fuciformis* (TF) is widely recognized as a natural ingredient with excellent water holding capacity and gelling properties [19]. As an edible fungus, TF exhibits anti-inflammatory and immunomodulatory activities and is rich in dietary fiber [20,21]. In general, dietary fiber enhances system viscosity through water absorption and swelling, and reinforces protein networks via physical filling effects. However, unlike other natural dietary fibers, TF is rich in highly branched acidic polysaccharides. Its molecular structure, characterized by abundant hydrophilic groups and flexible side chains, confers strong hydration capacity and high viscosity even at low concentrations, and enables the formation of weak gel-like structures in aqueous environments. Based on these structural characteristics, *Tremella fuciformis* polysaccharides may participate in protein network formation through spatial entanglement and intermolecular interactions, thereby enhancing structural continuity and water retention while providing mechanical support to the gel matrix.

In practical applications, TF is typically incorporated into food systems in the form of whole powder. For particulate ingredients, particle size influences dispersion behavior and integration within the protein matrix, ultimately affecting the balance between gel strength and sensory acceptance. Particle size reduction has been widely applied to improve the functional and textural performance of powdered ingredients in food systems [22,23,24,25]. For example, reducing the particle size of pumpkin powder added to yogurt improved its rheological properties and sensory acceptance [26]. Smaller psyllium husk (D_50_ = 259.50 µm) has been demonstrated to enhance the gel strength, viscosity, and moisture retention in surimi gels [27]. Mao et al. [28] also reported that the incorporation of smaller wheat bran particles (12.97 µm) facilitated the formation of a continuous gel matrix and reduced the bitterness of surimi gels. Therefore, optimizing the particle size of *Tremella fuciformis* powder (TFP) is critical for fully realizing its functional potential in soy yogurt. However, to date, relatively few studies have investigated how TFP particles of different sizes influence the gel properties of soy yogurt systems.

This study aims to investigate the influence of TFP particle size on the gel properties of soy yogurt, while elucidating the underlying structure–property relationships through intermolecular interaction analysis and microstructural observations.

## 2. Materials and Methods

### 2.1. Materials

*Tremella fuciformis* (polysaccharide 3.44%, moisture 1.69%, and ash 6.33%) was obtained from Gutian County, Fujian Province, China. Soymilk powder (protein 38.7%, fat 20.4%, total sugar 13.1% and moisture 3.3%) was purchased from Wuzhou Bingquan Industrial Shareholding Co., Ltd. (Wuzhou, Guangxi, China), and sucrose was purchased from Yunnan Dianpeng Sugar Co., Ltd. (Kunming, Yunnan, China). The fermentation starter was provided by the Fermentation Engineering Research Group at South China Agricultural University, including *Streptococcus thermophilus*, *Lactococcus lactis*, and *Lactobacillus plantarum*. All chemicals used were of analytical grade.

### 2.2. Preparation of Tremella fuciformis Powder (TFP)

Fresh *Tremella fuciformis* was soaked in clean water to remove impurities, followed by drying in an oven at 60 °C. The dried samples were then ground using a universal grinder until no large intact fragments remained, and further pulverized with a cryogenic superfine grinder to obtain TFP. The TFP was sequentially sieved through 100-, 200-, 300-, 500-, and 800-mesh filters to produce five different particle size fractions, designated as TFP_100_, TFP_200_, TFP_300_, TFP_500_, and TFP_800_, respectively.

### 2.3. Characterization of TFP

#### 2.3.1. Particle Size Analysis of TFP

Considering TFP would be applied to aqueous soymilk dispersions, the average particle size and particle size distribution of the TFP samples were measured in a wet measurement system using a laser particle size analyzer (Malvern Instruments, Malvern, Worcestershire, UK) following the method of Gong et al. [29] with minor modifications.

#### 2.3.2. Water Holding Capacity (WHC) Analysis of TFP

WHC was determined according to the method described by Zuo et al. [30]. TFP was mixed with 30 mL of distilled water in a centrifuge tube (M1). The samples were heated in a water bath at 100 °C for 15 min. After cooling to room temperature, the tubes were centrifuged at 6000 rpm for 15 min. The supernatant was removed, and then the residual solids were weighed (M2). The WHC was calculated as follows:

(1) $$\mathrm{WHC}=\frac{M2-M1}{M}$$

#### 2.3.3. Scanning Electron Microscopy (SEM) Observation of TFP

The morphology of TFP was observed using scanning electron microscopy (SEM, EVO MA 15, ZEISS, Oberkochen, Germany). The samples were sputter-coated with gold and observed at 200× magnification to capture micrographs.

### 2.4. Preparation and Characterization of Soy Yogurts

#### 2.4.1. Preparation of Soy Yogurts

Soymilk powder and sucrose were added to distilled water at concentrations of 5.5% and 7% (*w*/*v*), respectively, to prepare soymilk. TFP was then added to the soymilk at concentrations of 0%, 1.0%, 1.5%, and 2.0% (*w*/*v*), respectively. The soymilk was heated and stirred continuously at 90 °C for 30 min for pasteurization. After cooling to 42 °C, the starter fermentation was added and vortexed to mix well. The mixture was fermented at 37 °C for 18 h until reaching pH 4.5. After fermentation, all samples were refrigerated at 4 °C for 24 h. Finally, CK-SY (blank control yogurt) and TFP-SY (yogurt with TFP) were obtained accordingly.

#### 2.4.2. Particle Size Analysis of Soy Yogurts

The particle size distribution and D_[4,3]_ of the samples were determined using a Laser Particle Size Analyzer (Mastersizer 3000, Malvern Panalytical Ltd., Malvern, UK) equipped with a wet method particle size testing system.

#### 2.4.3. Zeta Potential in Soy Yogurts

The zeta potential of the samples was measured using a Zetasizer Nano ZS (Malvern Panalytical Ltd., UK) following the method of Mobasserfar et al. [31] with slight modifications. Prior to measurement, the samples were diluted 50 times. The measurements were performed at 25 °C. The refractive indices of the particles and water were 1.450 and 1.330, respectively, while the absorption rate was 0.8872.

#### 2.4.4. Intermolecular Forces in Soy Yogurts

According to Lv’s method [32] with slight modifications, the samples were dissolved in various solvents as follows: SA (0.06 mol/L NaCl), SB (0.6 mol/L NaCl), SC (0.6 mol/L NaCl and 1.5 mol/L Urea), SD (0.6 mol/L NaCl and 8 mol/L Urea), SE (1.0 mol/L NaOH). Two grams of yogurt samples were dispersed in different solvents in 50 mL centrifuge tubes. The samples were homogenized at 10,000 rpm for 2 min. Subsequently, the dispersions were centrifuged at 10,000 rpm for 15 min at 4 °C. Finally, the protein content of the supernatant was determined by the BCA method and presented as S1, S2, S3, S4, and S5, respectively. The protein solubility was calculated as follows:

(2) $$\mathrm{electrostatic}\;\mathrm{interactions}\;\left(\%\right)\;=\frac{S2-S1}{S5}\;\times \;100\%$$

(3) $$\mathrm{hydrogen}\;\mathrm{bond}\;\left(\%\right)=\frac{S3-S2}{S5}\times 100\%$$

(4) $$\;\mathrm{hydrophobic}\;\mathrm{interaction}\;\left(\%\right)=\frac{S4-S3}{S5}\times 100\%$$

(5) $$\mathrm{covalent}\;\mathrm{bonds}\;\left(\%\right)=\frac{S5-S4}{S5}\times 100\%$$

#### 2.4.5. SEM Analysis of Soy Yogurts

The soy yogurt samples were pre-frozen in a −80 °C freezer for 12 h and subsequently freeze-dried for 24 h. The samples were cut into 5 mm × 5 mm × 3 mm cubes. The samples were sputter-coated with gold and observed at 3000× magnification to capture micrographs.

#### 2.4.6. WHC of Soy Yogurts

WHC was determined according to the method described by Luo [33] with minor adaptations. An empty centrifuge tube was weighed and recorded as M. The yogurt was added to the tube and the total mass, M1, was measured. The tube was centrifuged at 4000 rpm for 20 min at 4 °C. The residues were weighed (M2) after supernatant removal. Each sample was measured in triplicate. The WHC was calculated as follows:

(6) $$\mathrm{WHC}\;(\%)=\frac{M2-M}{M1-M}\times 100\%$$

#### 2.4.7. Rheological Properties of Soy Yogurts

The rheological properties of the soy yogurt samples were measured using a MCR 502 Rheometer (Anton Paar, Graz, Austria) following the method of Bourouis et al. [34] with minor modifications. The soy yogurts were pressed between stainless steel plates (diameter = 40 mm, gap = 1 mm). The testing temperature was maintained at 25 °C for the measurement of apparent viscosity. The measurements were conducted at a constant frequency of 1 Hz, with a shear rate range from 0 to 100 s^−1^. The data were fitted to the Herschel–Bulkley model:

(7) $$\tau =\tau_{0}+\kappa {\left(\gamma^{.}\right)}^{n}$$

where τ is the shear stress (Pa); τ_0_ is the yield stress (Pa); κ is the consistency index (Pa·s^n^); γ^.^ is the shear rate (s^−1^); and n is the flow behavior index.

The dynamic viscoelastic parameters, including storage modulus (G′) and loss modulus (G″), were determined through oscillation measurements. The strain was fixed at 0.5% (within the linear viscoelastic region), and the frequency range was from 0.1 to 100 rad/s. The storage modulus (G′) and loss modulus (G″) were recorded.

### 2.5. Statistical Analysis

All experimental data were set up in triplicate and statistical results were expressed as mean ± standard deviation. Excel and GraphPad Prism 9 were used for plotting the raw data and drawing figures, while IBM SPSS Statistics 26.0 was employed for one-way analysis of variance (ANOVA). Significant differences were analyzed using Duncan’s method, and a significance level of *p* < 0.05 was adopted.

## 3. Results and Discussion

### 3.1. Characterization of TFP

**Figure 1:**
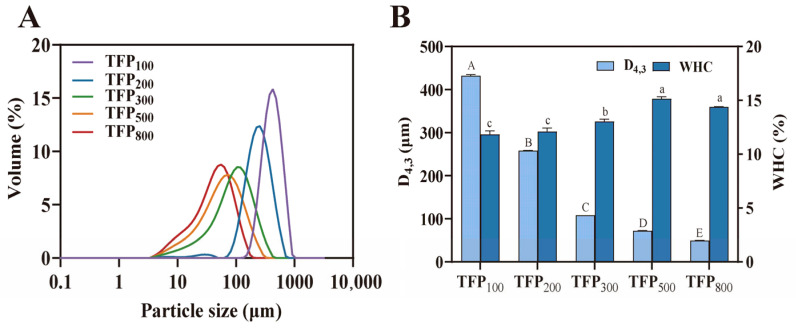
Particle size distribution (**A**) and WHC (**B**) of TFP. Different uppercase letters indicate significant differences in D_[4,3]_ among TFPs, while different lowercase letters indicate significant differences in WHC among TFPs (*p* < 0.05).

As shown in Figure 1, all TFP samples exhibited a unimodal particle size distribution, indicating uniform particle sizes within each sample. Moreover, a distinct leftward shift in the distribution curves was observed with decreasing particle size, demonstrating clear differences in the overall particle size distribution among the five TFP samples (Figure 1A). A significant decrease in D_[4,3]_ of TFP was recorded from TFP_100_ to TFP_800_ gradually (declining from 432.33 µm to 50.10 µm) (*p* < 0.05). Following mechanical grinding, intensified cell wall disruption and enlarged particle surface area were expected for these TFP samples [35].

WHC reflects the ability of a powder to hold water. As shown in Figure 1B, the WHC of TFP increased significantly with decreasing particle size, with TFP_500_ exhibiting the highest value of 15.13% (*p* < 0.05). Under mechanical forces, powder comminution increases the specific surface area, thereby expanding the water contact interface. Mechanical disruption of cellular structures exposes internal hydrophilic groups, facilitating greater hydrogen bonding with water molecules and thus enhancing the powder’s water retention capacity [36,37]. Furthermore, such processing releases *Tremella fuciformis* polysaccharides, which are intrinsically hygroscopic and capable of adsorbing and entrapping free water molecules [38].

**Figure 2:**
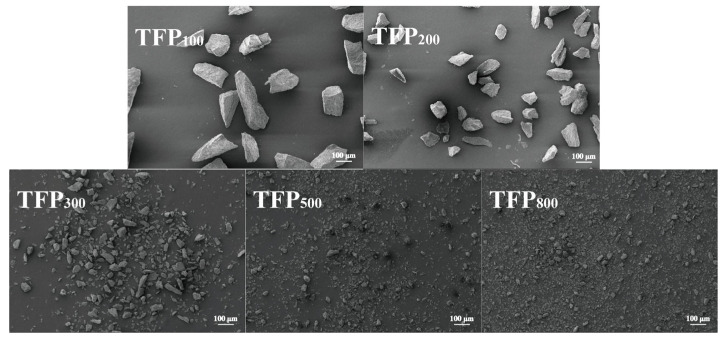
SEM images of TFP with different particle sizes (200×).

As shown in Figure 2, SEM observations revealed distinct morphological differences among the TFP samples. The unprocessed raw SEM images are available in the Supplementary Materials (Figure S1). The particles of TFP_100_ and TFP_200_ were relatively large, exhibiting predominantly elongated, tapered, and irregular shapes while maintaining high structural integrity with minimal debris. TFP_300_ showed a marked reduction in particle size, accompanied by increased fragmentation and a discernible shift toward more spherical morphologies. Notably, TFP_500_ and TFP_800_ comprised substantially finer, near-spherical particles, alongside a heightened density of debris and localized agglomerates.

### 3.2. Characterization of Soy Yogurts

#### 3.2.1. Effects of TFP Particle Size and Concentration on Zeta Potential of Soy Yogurts

**Figure 3:**
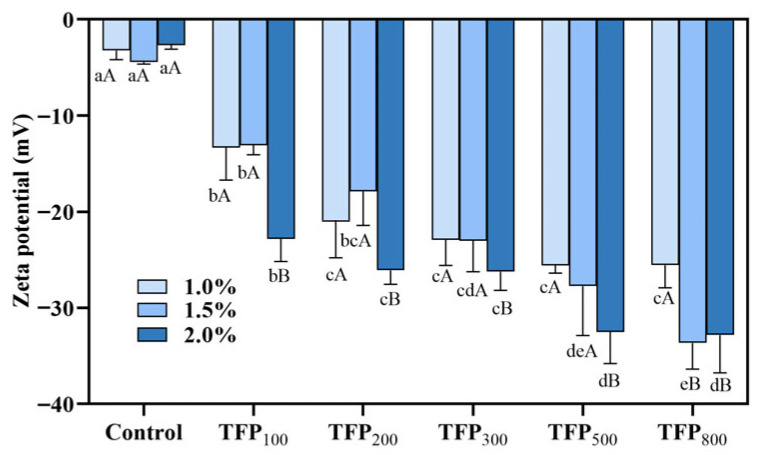
Zeta potential of soy yogurt with addition of TFP with different particle sizes at various concentrations. Different uppercase letters indicate significant differences among TFP-SYs with the same particle size at varying concentrations, while different lowercase letters indicate significant differences among TFP-SYs with different particle sizes under the same concentration (*p* < 0.05).

As shown in Figure 3, all samples exhibited negative zeta potential values. TFP-SY displayed a significantly higher absolute zeta potential than CK-SY (*p* < 0.05). The absolute zeta potential generally increased with decreasing TFP particle size. At a given particle size, samples containing 2% TFP showed higher absolute values compared to other concentrations.

The negative zeta potential of CK-SY may be attributed to the presence of net negative charges on the soy protein surfaces. At pH around 4.5, uronic acid residues (pKa 3–4) in the *Tremella fuciformis* polysaccharides dissociate into negatively charged -COO^−^ groups, thereby increasing the number of negatively charged groups in the soy yogurt system [39,40]. Increasing the TFP concentration introduces more of these charged groups, thereby increasing the overall negative charge density. Furthermore, when TFP particle size is reduced, the increased specific surface area facilitates greater exposure of uronic acid groups, which may lead to a higher surface charge and enhanced electrostatic repulsion [41]. These modifications in surface charge may be associated with changes in gel behavior during acidification.

#### 3.2.2. Effects of TFP Particle Size and Concentration on Intermolecular Interactions of Soy Yogurts

**Figure 4:**
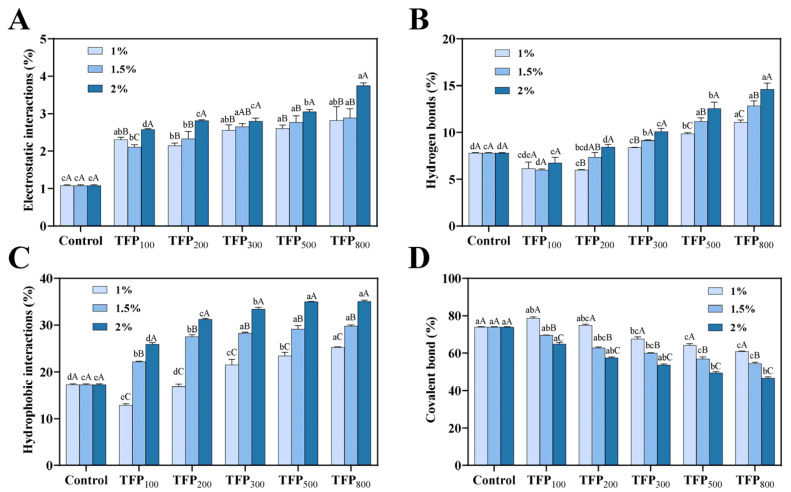
Intermolecular forces of soy yogurt with addition of TFP with different particle sizes at various concentrations: (**A**) electrostatic interactions; (**B**) hydrogen bonds; (**C**) hydrophobic interactions; (**D**) covalent bonds. Different uppercase letters indicate significant differences among TFP-SYs with the same particle size at varying concentrations, while different lowercase letters indicate significant differences among TFP-SYs with different particle sizes under the same concentration (*p* < 0.05).

Figure 4 illustrates the intermolecular forces in soy yogurt, including electrostatic interactions, hydrogen bonds, hydrophobic interactions, and covalent bonds. The incorporation of TFP was observed to significantly alter these intermolecular forces (*p* < 0.05). The relative contributions of these interactions in soy yogurt generally followed the order: covalent bonds > hydrophobic interactions > hydrogen bonds > electrostatic interactions. The primary stabilizing forces maintaining the gel conformation of soy yogurt were identified as covalent bonds and hydrophobic interactions, followed by hydrogen bonds and electrostatic interactions.

As shown in Figure 4A, electrostatic interactions accounted for a relatively low proportion of the total intermolecular forces in soy yogurt. They gradually strengthened due to the increase in TFP concentration and the reduction in particle size, a trend that aligned with the zeta potential measurements (Figure 3). This enhancement can be attributed to the introduction of negatively charged polysaccharides from TFP, which increase overall electrostatic repulsion. Meanwhile, the negatively charged TFP particles can interact with positively charged regions on the proteins (even when close to its isoelectric point), forming electrostatic bonds [42].

In Figure 4B, hydrogen bond content decreased with the addition of larger TFP particles (i.e., TFP_100_ and TFP_200_). However, a significant enhancement was observed for the finer particle sizes (i.e., TFP_300_ and below) at all concentrations (*p* < 0.05). The proportion of hydrogen bonds was generally increased with higher TFP concentrations, except for TFP_100_. This phenomenon may be attributed to the non-uniform distribution and sedimentation of large TFP particles, which disrupted the original hydrogen bond network in soy yogurt matrix [28]. However, smaller TFP particles remain predominantly suspended, allowing the hydroxyl and carboxyl groups on their polysaccharide chains to actively contribute to hydrogen bond formation [43]. Increased hydrogen bonding is commonly associated with improved gel strength [44]. Although hydrogen bonds and electrostatic interactions contribute less to the overall gel structure as compared to hydrophobic interactions, they remain critical for stabilizing secondary structures (e.g., α-helices) of proteins to enable gelation [45].

As shown in Figure 4C, hydrophobic interactions in soy yogurt played an important role in soy protein gelation, accounting for around 10 to 20%. Generally, an increasing trend was observed with the addition of TFP at 1% with smaller particle size, except for the samples with the addition of TFP_100_ and TFP_200_, which showed a slight reduction or remained stable compared to CK-SY. At higher concentrations (1.5% and 2%), similarly, the proportion of hydrophobic interactions in TFP-SY was elevated with the addition of TFP with smaller particle size. Moreover, the hydrophobic interactions of soy yogurt were progressively enhanced with increasing TFP concentration from 1% to 2%. At low concentrations, the considerable steric hindrance of large particles reduced protein aggregation [32]. However, the enhanced water binding capacity of smaller TFP particles was observed to absorb surrounding water from soy proteins, resulting in a localized “concentrated” state. This created a relatively hydrophobic environment, which promoted protein unfolding and exposed hydrophobic groups, thereby strengthening hydrophobic interactions [46]. As the concentration increased, the intensified concentration effect further amplified hydrophobic interactions, ultimately facilitating the formation of a more compact and structurally stable gel network.

The covalent bonds were the main force maintaining the soy yogurt gel, and the corresponding proportion was as high as 73.90% for the control sample (Figure 4D). The covalent bond proportion in soy yogurt was significantly reduced by TFP addition (*p* < 0.05), except for TFP_100_ and TFP_200_ at 1%. Similar reductions in covalent interactions were observed in soy protein isolate gels incorporated with konjac gum and curdlan gum [47]. Moreover, the covalent bond content was further reduced with decreasing particle size of TFP, with the lowest covalent bond proportion observed in 2% TFP_800_-SY. This trend may be related to the steric hindrance effect of particles, which reduced the efficiency of covalent cross-linking between protein molecules. Although the covalent bond proportion decreased, it remained the predominant structural component of the gel network, providing rigid structural support to the system.

#### 3.2.3. Effects of TFP Particle Size and Concentration on Particle Size of Soy Yogurts

**Figure 5:**
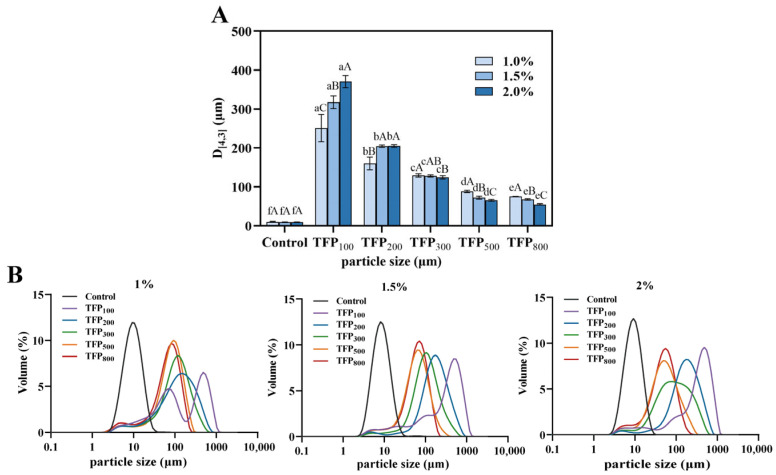
Particle size distribution of soy yogurt with addition of TFP with different particle sizes at various concentrations. (**A**) D_[4,3]_; (**B**) particle size distribution. Different uppercase letters indicate significant differences among TFP-SYs with the same particle size at varying concentrations, while different lowercase letters indicate significant differences among TFP-SYs with different particle sizes under the same concentration (*p* < 0.05).

The D_[4,3]_ and particle size distribution of soy yogurt with and without TFP addition are displayed in Figure 5. The D_[4,3]_ in soy yogurt without TFP addition was 10 µm (Figure 5A), with a monomodal distribution (Figure 5B). Following TFP addition, the D_[4,3]_ of soy yogurt significantly increased with a shoulder in addition to the main peak in their particle size distribution (*p* < 0.05). Furthermore, the D_[4,3]_ decreased with the addition of TFP with smaller particle size. Nevertheless, concentration effects of various TFPs showed distinct discrepancies in the D_[4,3]_ of soy yogurt. Higher D_[4,3]_ of the yogurts with the addition of TFP_100_ and TFP_200_ was found at higher TFP concentrations, whereas the D_[4,3]_ of yogurts containing TFP_300_, TFP_500_, and TFP_800_ was observed to decrease as TFP concentration increased.

As expected, the formation of large protein aggregates was promoted by the addition of TFP as a thickener. The water-absorbing behavior of TFP increases the likelihood of protein interaction with each other, leading to protein structure unfolding, exposure of hydrophobic groups, and enhanced intermolecular interactions, thereby promoting the formation of large aggregates [48]. From TFP_300_ onward, the D_[4,3]_ value of soy yogurt demonstrates an inverse trend with concentration. This may be attributed to the smaller particle size of TFP, which facilitates better dispersion in the solution. Nevertheless, excessive dispersion, volume expansion upon water absorption, and enhanced electrostatic repulsion collectively inhibit the formation of large aggregates. It was reported that the creaminess and consistency of reduced-fat yogurt are influenced by particle size, whereby smaller particles (below 150 µm) are associated with a smoother texture and creamier appearance [49]. Notably, at a concentration of 1–2%, the average particle size of TFP_500_-SY and TFP_800_-SY remained below 100 µm. Consequently, soy yogurts incorporating smaller TFP particles may achieve enhanced creaminess [50].

#### 3.2.4. Effects of TFP Particle Size and Concentration on SEM of Soy Yogurts

**Figure 6:**
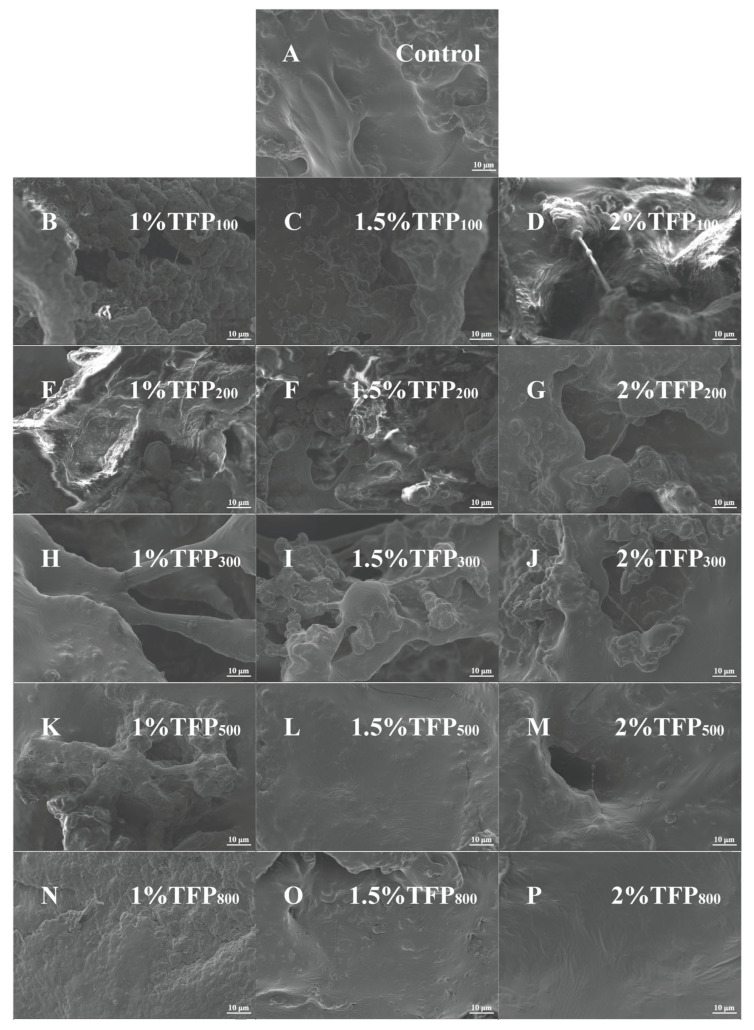
SEM images of soy yogurt with and without addition of TFP with different particle sizes at various concentrations (3000×).

As shown in Figure 6, the microstructure of soy yogurts with or without TFP addition was observed using SEM. The unprocessed raw SEM images are available in the Supplementary Materials (Figure S2). In the absence of TFP, the soy yogurt gel exhibited a loose, porous, and rough three-dimensional network structure, with distinctly visible cavities (Figure 6A). These cavities have been reported to be associated with water-rich regions within the gel matrix and may be linked to structural heterogeneity [51]. Upon the addition of TFP, both the number and size of these cavities showed noticeable variation. When TFP_100_ and TFP_200_ were incorporated, the network appeared less continuous (Figure 6B–G) than the control. The gel network remained irregular and fragmented with a more heterogeneous morphology, which may be related to the presence of relatively large TFP particles that interfere with the spatial arrangement of the protein network. With reduced TFP particle size and increased concentration, the microstructure gradually became denser and more compact, with fewer visible cavities and a relatively smoother morphology (Figure 6H–P). Furthermore, the continuity of the soy protein network was improved, and the cavities became less pronounced. Notably, at 1.5% and 2% concentration, TFP_500_-SY and TFP_800_-SY exhibited a more integrated morphology, in which particulate structures were embedded within the protein matrix (Figure 6L,M,O,P). It is speculated that TFP exhibited enhanced water binding capacity, which may influencing moisture distribution within the gel matrix and reduce the prominence of visible cavities [52]. The dehydration effect exhibited by TFP may increase the relative protein concentration, which in turn may affect unfolding and aggregation. This modification may strengthen intermolecular interactions between proteins, which is consistent with the increased hydrophobic interactions (Figure 4C) and contribute to the formation of a homogeneous and compact microstructure [53,54]. Furthermore, TFP with appropriate size has been reported to be not only incorporated as fillers within the protein network structure to enhance gel strength but also to potentially participate in network formation [55], both of which may contribute to the observed morphological differences in the protein gels.

#### 3.2.5. Effects of TFP Particle Size and Concentration on WHC of Soy Yogurts

**Figure 7:**
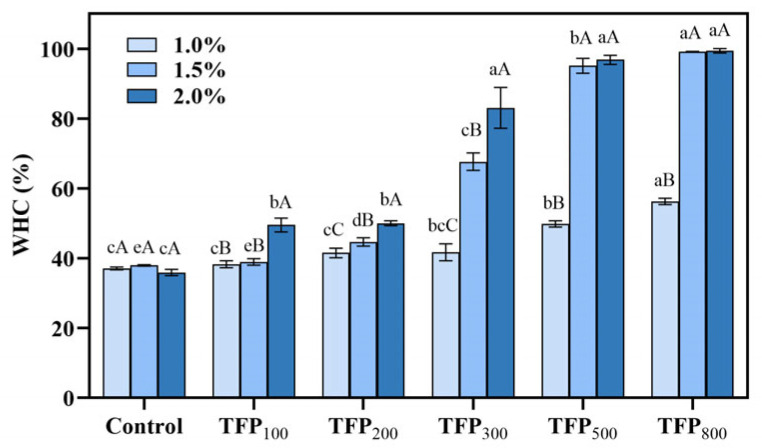
WHC of soy yogurt with addition of TFP with different particle sizes at various concentrations. Different uppercase letters indicate significant differences among TFP-SYs with the same particle size at varying concentrations, while different lowercase letters indicate significant differences among TFP-SYs with different particle sizes under the same concentration (*p* < 0.05).

As demonstrated in Figure 7, the WHC of soy yogurt without TFP was as low as 37%. A significant improvement in WHC was observed following TFP addition (*p* < 0.05), correlating with increased TFP concentrations. Upon 1% TFP addition, the enhancement in WHC was limited with the addition of TFP with large particle size, but a significant increase was recorded with TFP_500_ and TFP_800_ (*p* < 0.05). In contrast, more pronounced increases in WHC were achieved at higher TFP concentrations (i.e., 1.5% and 2%), particularly with TFP_300_, TFP_500_, and TFP_800_, with WHC reaching 99.12% and 99.44% upon addition of 2% TFP_500_ and TFP_800_, respectively. Similar high WHC values (95–99%) were reported by Gomes et al. [50] following the incorporation of different soluble dietary fibers into yogurt.

TFP itself exhibits strong water adsorption capacity due to the presence of hydrophilic groups (e.g., hydroxyl and carboxyl groups), which are capable of interacting with water molecules and stabilizing them within the gel matrix [56]. Smaller TFP particles may penetrate or fill the protein gel network more effectively, exposing additional hydrophilic groups and promoting intermolecular interactions (e.g., enhanced hydrogen bonding) [57], thereby improving water retention. Furthermore, insoluble dietary fibers in TFP are suggested to reinforce gel strength, mitigate network collapse, and immobilize water within porous gel structures [58].

#### 3.2.6. Effects of TFP Particle Size and Concentration on Rheological Properties of Soy Yogurts

**Figure 8:**
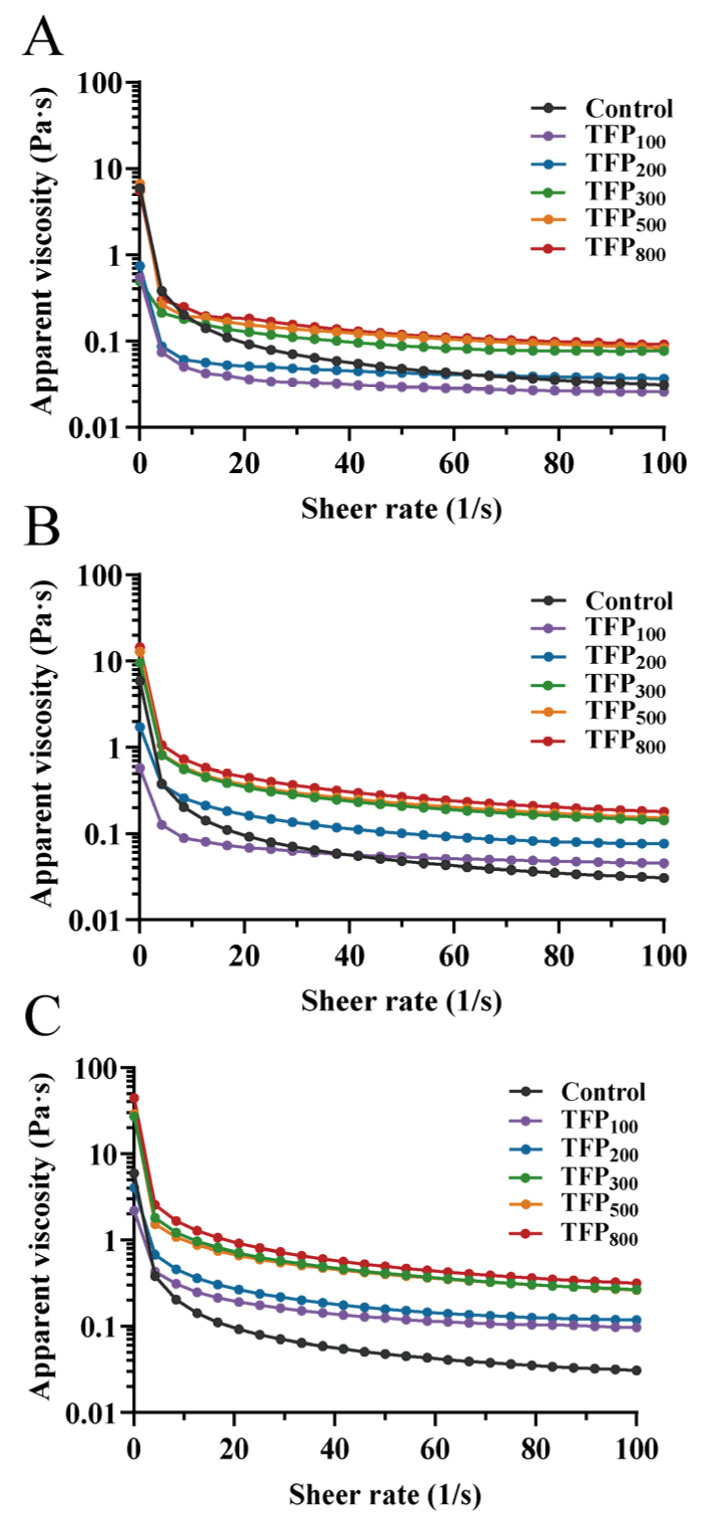
Apparent viscosity of soy yogurt with addition of TFP with different particle sizes at various concentrations. (**A**) 1%; (**B**) 1.5%; (**C**) 2%.

The apparent viscosity of soy yogurts is shown in Figure 8, and the flow behavior of soy yogurt is simulated using the Herschel–Bulkley model with the corresponding parameters presented in Table 1.

Yield stress (τ_0,_ i.e., the minimum stress required to initiate flow) was significantly reduced in soy yogurts with 1.0% and 1.5% TFP addition at all particle sizes compared to CK-SY. However, with 2% TFP addition, the τ_0_ of TFP_300_-SY, TFP_500_-SY, and TFP_800_-SY was significantly higher than that of CK-SY (*p* < 0.05). At lower concentrations (1% or 1.5%), the original protein structure was presumed to be disrupted by the sedimentation of large TFP particles, leading to reduced resistance to external deformation. In contrast, with 2% TFP, independent gel-phase formation or synergistic crosslinking between TFP and soy proteins was proposed to partially counteract these destabilizing effects. The consequent increase in τ_0_ reflects enhanced stability within the gel network, imparting greater resistance to shear and external stress. This improvement in the stability of the gel network effectively minimizes phase separation and syneresis, thereby extending the product’s shelf stability.

It was observed that all samples exhibited the shear-thinning behavior in Figure 8. As the shear rate increased to disrupt the stabilizing forces, the long-chain molecules (mostly proteins) were stretched along the direction of motion under shear forces. The dissociation rate of proteins exceeded the entanglement rate, resulting in a reduction in apparent viscosity [59]. The increase in the consistency index (κ) is considered indicative of the formation of protein-polysaccharide complexes, which contribute to cross-linking networks and viscosity enhancement [60]. At 1% TFP addition, only the κ of TFP_800_-SY was higher than that of CK-SY, whereas at 2%, all TFP-SY samples exhibited higher κ than CK-SY. This may be attributed to the enhanced intermolecular friction during fluid flow caused by increased concentrations, leading to viscosity alterations in soy yogurt. Similarly, the κ of soy yogurt was observed to increase with decreasing TFP particle size, with TFP_800_-SY showing the highest κ and TFP_100_-SY the lowest at the same concentration. Smaller particle size TFP demonstrated enhanced water retention capacity (Figure 1B). Following water absorption, volume expansion of the hydrated particles restricts intermolecular mobility, thereby increasing the viscosity of soy yogurt.

All fluids exhibited a flow behavior index (n) < 1, demonstrating pseudoplastic behavior. Lower n values correlated with stronger pseudoplasticity and more pronounced shear-thinning tendencies. The n value of soy yogurt was reduced with increasing TFP concentration and decreasing particle size. These trends implied that higher concentrations of finer TFP particles enhanced pseudoplasticity, contributing to a smooth mouthfeel during mastication and stable viscosity recovery post-swallowing [61].

**Figure 9:**
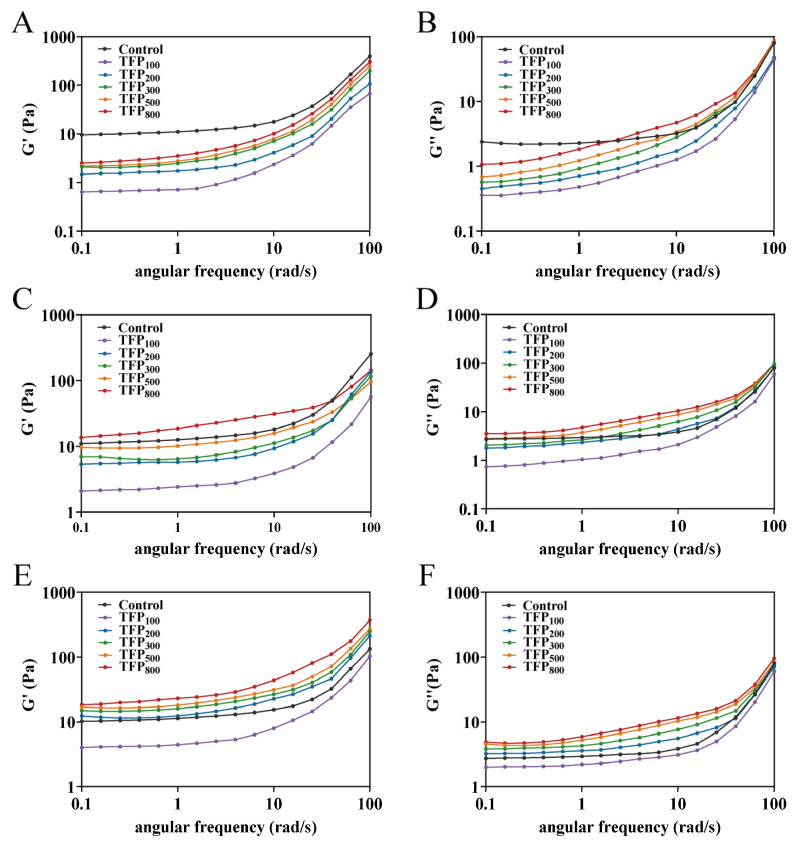
Rheological properties of soy yogurt with addition of TFP with different particle sizes at various concentrations. (**A**,**B**): G′ and G″ at 1%; (**C**,**D**): G′ and G″ at 1.5%; (**E**,**F**): G′ and G″ at 2%.

The rheological properties of soy yogurt were analyzed by quantifying storage modulus (G′) and loss modulus (G″), which represented the elastic and viscous behavior of gel systems, respectively [62]. As shown in Figure 9, all soy yogurts exhibited higher G′ than G″, indicating their gel-like behavior. Notably, the gels dominated by covalent bonds typically lack frequency dependence due to stable crosslinking, whereas those governed by non-covalent interactions display pronounced frequency sensitivity [63]. The observation of frequency dependence of G′ and G″ in all samples suggested that non-covalent interactions may contribute importantly to the structural integrity of soy yogurt gels.

The rheological properties of soy yogurt were largely influenced by both TFP concentration and its particle size. When comparing rheological data at a frequency of 0.1 rad/s, the G′ and G″ of soy yogurt were reduced upon 1% TFP addition, while this reduction effect was clearly diminished at 1.5%. Instead, at 2%, the G′ and G″ of soy yogurt were all higher than those of the control sample, except TFP_100_-SY. At the same concentration, the G′ and G″ of soy yogurt both showed higher values upon addition of TFP with smaller particle size compared to large particle size TFP. Notably, the effect of TFP_800_-SY was the most pronounced.

The incorporation of large TFP particles (TFP_100_ and TFP_200_) failed to improve the viscoelasticity of soy yogurt and even substantially reduced it. This arose from the heterogeneous distribution of large particles during soy yogurt gelation, resulting in localized aggregation and discontinuous network structures (as observed in the microstructure, Figure 6B–J). The large particles act as structural defects within the gel matrix, readily serving as fracture initiation sites when external stress is applied [64]. Moreover, the steric hindrance contributed by large particles (as large as 370 µm in Figure 5) in soy yogurt impeded protein aggregation, thereby reducing intermolecular interactions and yielding less compact protein conformations with diminished viscoelasticity. In comparison, reducing TFP particle size facilitated the formation of smaller aggregates (<100 µm) in soy yogurt, enabling more homogeneous protein clustering. Smaller-sized particles of TFP exhibit stronger water absorption, competing with proteins for the environmental moisture. This leads to a “concentrated environment” for proteins, enhancing protein–protein interactions, which is consistent with the results on intermolecular interactions in Figure 4. The increases in G′ and G″ upon introducing TFP_300_, TFP_500_, and TFP_800_ were also associated with the strengthening of non-covalent interactions (as shown in Figure 4). Meanwhile, smaller-sized TFP can be more easily incorporated into the protein network structure, functioning as a “filler” to enhance gel stability [65]. Through its inherent water-absorbing capacity, smaller-particle-size TFP additionally immobilizes free water molecules, thereby strengthening the protein matrix. This process enhances matrix continuity and fracture resistance, which was corroborated by SEM observations in Figure 6K–P. This is also supported by McCann et al. [66], whereby incorporating carrot cell wall particles into yogurt could occupy voids within protein networks to reinforce gel hardness.

At lower concentrations, TFP may be insufficient to form effective coacervates with soy protein. This reduces interactions between proteins and diminishes both the continuity of the soy yogurt network structure and its viscoelasticity. In contrast, a 2% addition of TFP exerts a more positive effect on the viscoelastic properties of soy yogurt. At higher concentrations, the swollen particles dominate the rheological properties. By absorbing water within the continuous phase, these particles expand and exert pressure on the protein structure by filling its pores. This promotes interactions both among proteins and between TFP and proteins, thereby facilitating the formation of a stronger gel matrix [67]. Furthermore, the increased TFP concentration significantly enhances the WHC of soy yogurt. The immobilized water can act as a filler within the gel, increasing the internal resistance and further contributing to the improved viscoelastic properties. In summary, a stronger elastic structure in soy yogurt is promoted by the synergistic combination of smaller-sized TFP and high concentration, making it more resistant to external deformation.

## 4. Conclusions

This study investigated the effects of TFP particle size on the gel properties of soy yogurt. The gel structure of soy yogurt was relatively fragile, characterized by a three-dimensional network containing numerous water channels. Therefore, soy yogurt exhibited low gel strength, making it prone to structural collapse and syneresis. A substantial alteration of the network structure was observed resulting from the incorporation of TFP. Large particles tended to hinder normal protein cross-linking and network formation, while gravitational settling of larger particles may further disrupt the original spatial arrangement of proteins. In contrast, reducing particle size significantly increased the WHC, apparent viscosity, and viscoelastic properties of soy yogurt. As particle size decreased, TFP particles more effectively filled pores within the protein matrix and may have exerted steric pressure on the protein structure through swelling-induced volumetric expansion. This process may contribute to protein densification and closer intermolecular contact, potentially strengthening protein–protein interactions and improving gel matrix continuity, leading to a more compact and stable three-dimensional network. Moreover, increasing the TFP concentration further enhanced its positive effect on soy yogurt gel properties. Overall, this study extends established interaction and filler effect concepts to plant-based yogurt applications. TFP particle size influenced its integration into the soy protein network and the resulting microstructural organization. This result suggests an association between particle size-regulated network remodeling and improved gel performance, supporting the development of stable plant-based yogurt products.

However, the reduction in TFP particle size was achieved through mechanical processing. In addition to decreasing particle size, this treatment may have induced molecular-level changes, such as altering polysaccharide chain length, cell structure disruption, and functional group exposure. These processing-induced changes may act synergistically with the particle size effect, making it difficult to accurately determine the role of TFP in soy yogurt. Therefore, the mechanism by which TFP improves soy yogurt gels still requires further verification. Future work will combine molecular characterization with yogurt model systems to elucidate interaction pathways based on solution and gel behaviors. In this way, it is expected to provide a more reliable basis for structural regulation of plant-based yogurts.

## Figures and Tables

**Table 1:** Rheological parameters of the flow curves of the yogurt containing TFP.

	Sample	τ_0_	κ (Pa·s^n^)	n
	Control	0.502 ± 0.026 ^aA^	0.494 ± 0.027 ^bA^	0.344 ± 0.013 ^dA^
1%	TFP_100_	0.091 ± 0.007 ^cB^	0.054 ± 0.013 ^cC^	0.834 ± 0.066 ^abcA^
	TFP_200_	0.047 ± 0.012 ^dC^	0.091 ± 0.008 ^cC^	0.801 ± 0.022 ^aA^
	TFP_300_	0.233 ± 0.021 ^bB^	0.251 ± 0.026 ^aC^	0.726 ± 0.020 ^abA^
	TFP_500_	0.248 ± 0.013 ^bC^	0.452 ± 0.011 ^bC^	0.629 ± 0.001 ^cA^
	TFP_800_	0.266 ± 0.020 ^bB^	0.524 ± 0.090 ^abC^	0.614 ± 0.032 ^bcA^
1.5%	TFP_100_	0.020 ± 0.007 ^cC^	0.145 ± 0.011 ^dB^	0.744 ± 0.015 ^aB^
	TFP_200_	0.116 ± 0.012 ^bcB^	0.661 ± 0.021 ^bB^	0.517 ± 0.006 ^bB^
	TFP_300_	0.255 ± 0.067 ^bB^	1.738 ± 0.008 ^aB^	0.452 ± 0.010 ^cdB^
	TFP_500_	0.491 ± 0.145 ^aB^	1.689 ± 0.083 ^aB^	0.470 ± 0.009 ^cB^
	TFP_800_	0.461 ± 0.193 ^aB^	2.283 ± 0.349 ^abB^	0.443 ± 0.020 ^dB^
2%	TFP_100_	0.354 ± 0.049 ^fA^	0.569 ± 0.000 ^deA^	0.601 ± 0.000 ^aB^
	TFP_200_	0.376 ± 0.047 ^eA^	1.123 ± 0.000 ^dA^	0.492 ± 0.000 ^aC^
	TFP_300_	0.657 ± 0.063 ^cA^	4.312 ± 0.029 ^cA^	0.446 ± 0.002 ^bC^
	TFP_500_	1.160 ± 0.009 ^bA^	3.258 ± 0.030 ^bA^	0.389 ± 0.001 ^cB^
	TFP_800_	1.695 ± 0.091 ^aA^	5.847 ± 0.091 ^aA^	0.351 ± 0.001 ^dC^

Different uppercase letters indicate significant differences among TFP-SYs with the same particle size at varying concentrations, while different lowercase letters indicate significant differences among TFP-SYs with different particle sizes under the same concentration (*p* < 0.05).

## Data Availability

Data are available from the corresponding author upon reasonable request.

## Supplementary Materials

The following supporting information can be downloaded at: https://www.mdpi.com/article/10.3390/foods15061000/s1, Unprocessed raw SEM images supporting this study are available as Supplementary Figures S1 and S2. Figure S1: Unprocessed raw SEM images of TFP samples; Figure S2: Unprocessed raw SEM images of soy yogurt samples.

## References

[1] Ma Y., Lee S.H., Guk M., Cho S., Kim Y. (2025). Enhancing soy yogurt texture and functionality with mealworm protein. J. Food Compos. Anal..

[2] Sá A.G.A., Moreno Y.M.F., Carciofi B.A.M. (2020). Plant proteins as high-quality nutritional source for human diet. Trends Food Sci. Technol..

[3] Li S., Wang C., Dai Y., Dai J., Wang W. (2025). Novel technologies, effects and applications of modified plant proteins by Maillard reaction and strategies for regulation: A review. Food Res. Int..

[4] Dhakal D., Younas T., Bhusal R.P., Devkota L., Henry C.J., Dhital S. (2023). Design rules of plant-based yoghurt-mimic: Formulation, functionality, sensory profile and nutritional value. Food Hydrocoll..

[5] Cui L., Chang S.K.C., Nannapaneni R. (2021). Comparative studies on the effect of probiotic additions on the physicochemical and microbiological properties of yoghurt made from soymilk and cow’s milk during refrigeration storage (R2). Food Control.

[6] Arpitha S.R., Sasi M., Kumar S., Yeasin M., Harun M., Sethi S., Kumari S., Nain L.R., Amirtham D., Sachdev A. (2025). Optimizing single and co-culture soymilk fermentation using *Weissella* probiotics for improved nutritional and sensory quality. Food Biosci..

[7] Lin X., Cao Z., Zhang J., Mu G., Jiang S. (2024). Characteristics of the Mixed Yogurt Fermented from Cow-Soy Milk in the Presence of Transglutaminase. Foods.

[8] Grasso N., Alonso-Miravalles L., O’Mahony J.A. (2020). Composition, Physicochemical and Sensorial Properties of Commercial Plant-Based Yogurts. Foods.

[9] Ren W., Liang H., Liu S., Li Y., Chen Y., Li B., Li J. (2024). Formulations and assessments of structure, physical properties, and sensory attributes of soy yogurts: Effect of carboxymethyl cellulose content and degree of substitution. Int. J. Biol. Macromol..

[10] Rezaei R., Khomeiri M., Kashaninejad M., Aalami M., Mazaheri-Tehrani M. (2018). Steady and dynamic rheological behaviour of frozen soy yogurt mix affected by resistant starch and β-glucan. Int. J. Food Prop..

[11] Kong X., Xiao Z., Du M., Wang K., Yu W., Chen Y., Liu Z., Cheng Y., Gan J. (2022). Physicochemical, Textural, and Sensorial Properties of Soy Yogurt as Affected by Addition of Low Acyl Gellan Gum. Gels.

[12] Himashree P., Sengar A.S., Sunil C.K. (2022). Food thickening agents: Sources, chemistry, properties and applications—A review. Int. J. Gastron. Food Sci..

[13] Maruyama S., Streletskaya N.A., Lim J. (2021). Clean label: Why this ingredient but not that one?. Food Qual. Prefer..

[14] Vicent V. (2024). Influence of banana powder on proximate composition, physicochemical and rheological properties of soy yoghurt. Appl. Food Res..

[15] Zhang R., Zhang J., Zhang Y., Zhang H., Cao Y., Xu W. (2025). Effects of *Citri Reticulatae Pericarpium* on the physicochemical, rheological, and sensory properties of soy yogurt. LWT.

[16] Mykhalevych O., Stapelfeldt H., Marini F., Bro R. (2025). Chemometric insights into milk-carrageenan breaking and gel strength. Food Hydrocoll..

[17] Khushbu S., Sunil C.K., Chidanand D.V., Jaganmohan R. (2019). Effect of particle size on compositional, structural, rheological, and thermal properties of shallot flour as a source of thickening agent. J. Food Process Eng..

[18] Huang L., Cai Y., Liu T., Zhao X., Chen B., Long Z., Zhao M., Deng X., Zhao Q. (2019). Stability of emulsion stabilized by low-concentration soybean protein isolate: Effects of insoluble soybean fiber. Food Hydrocoll..

[19] Xu J., Zou Y., Guo L., Lin J., Jiang Z., Zheng Q. (2023). Rheological and microstructural properties of polysaccharide obtained from the gelatinous *Tremella fuciformis* fungus. Int. J. Biol. Macromol..

[20] Deng W., Wu L., Xiao Z., Li Y., Zheng Z., Chen S. (2023). Structural Characterization and Anti-Inflammatory Activity of Polysaccharides from *Tremella fuciformis* on Monosodium Urate-Stimulated RAW264.7 Macrophages. Foods.

[21] Huang T.-Y., Yang F.-L., Chiu H.-W., Chao H.-C., Yang Y.-J., Sheu J.-H., Hua K.-F., Wu S.-H. (2022). An Immunological Polysaccharide from *Tremella fuciformis*: Essential Role of Acetylation in Immunomodulation. Int. J. Mol. Sci..

[22] Kieserling K., Vu T.M., Drusch S., Schalow S. (2019). Impact of pectin-rich orange fibre on gel characteristics and sensory properties in lactic acid fermented yoghurt. Food Hydrocoll..

[23] Song Z., Dai H., Bo L., Song C., Liu X., Ren J. (2024). Set yogurt incorporated with insoluble dietary fiber maintains non-sedimentation: Combined alkaline hydrogen peroxide modification and high-pressure homogenization process. Food Sci. Technol..

[24] Wang T., Chen J., Zhong Y., Xu D., Ren D. (2025). Investigating the role of lemon peel fiber in the casein gelation mechanism of low-fat yogurt. Food Hydrocoll..

[25] Zheng H., Wang W., Deng K., Wang L. (2025). Enhancement of dietary fiber yoghurt using high-pressure homogenized bacterial cellulose: Effects on microstructure, water distribution, and sensory properties. Int. J. Biol. Macromol..

[26] Gavril Rațu R.N., Cârlescu P.M., Veleșcu I.D., Arsenoaia V.N., Stoica F., Stănciuc N., Aprodu I., Constantin O.E., Râpeanu G. (2024). The development of value-added yogurt based on pumpkin peel powder as a bioactive powder. J. Agric. Food Res..

[27] Zhu S., Jin Y., Wei C., Yang Q., Wei Z., Song H., Liu S., Ding Y., Zhou X. (2024). Characterisation of psyllium husk with different sizes and their effects on gel properties of surimi. Int. J. Food Sci. Technol..

[28] Mao Y., Zhang Y., Li T., Chen Y., Wang Z., Jin W., Shen W., Li J. (2025). Insight into the mechanism of gel properties, microstructure and flavor of surimi gels improved by wheat bran with different particle sizes. Food Res. Int..

[29] Gong P., Huang Z., Guo Y., Wang X., Yue S., Yang W., Chen F., Chang X., Chen L. (2022). The effect of superfine grinding on physicochemical properties of three kinds of mushroom powder. J. Food Sci..

[30] Zuo S., Zhang R., Zhang Y., Liu Y., Wang J. (2018). Studies on the Physicochemical and Processing Properties of Tremella fuciformis Powder. Int. J. Food Eng..

[31] Mobasserfar R., Shiri A., Mofid V., Shahidi Noghabi M., Gharibzahedi S.M.T. (2024). Grape pomace high-methoxyl pectin: A new prebiotic stabilizer for low-fat synbiotic yogurt gels—Optimization and characterization. Int. J. Biol. Macromol..

[32] Lv Y., Tang T., Xu L., Wang J., Su Y., Li J., Gu L., Zhang M., Yang Y., Chang C. (2022). Influence of soybean dietary fiber with varying particle sizes and transglutaminase on soy protein isolate gel. Food Res. Int..

[33] Luo J., Liu S., Lu H., Wang Y., Chen Q., Shi Y. (2023). Improvement of kefir fermentation on rheological and microstructural properties of soy protein isolate gels. Food Res. Int..

[34] Bourouis I., McClements D.J., Chen C., Li H., Pang Z., Liu X. (2024). Formulations and evaluations of structural and physico-chemical properties of soy yogurts: Effect of incorporating soy protein isolate/chitosan complexed microgels. Food Sci. Technol..

[35] Zhao X., Liu H., Zhang X., Ao Q. (2018). Effect of pressure grinding technology on the physicochemical and antioxidant properties of *Tremella aurantialba* powder. J. Food Process. Preserv..

[36] Zhong C., Zu Y., Zhao X., Li Y., Ge Y., Wu W., Zhang Y., Li Y., Guo D. (2016). Effect of superfine grinding on physicochemical and antioxidant properties of pomegranate peel. Int. J. Food Sci. Technol..

[37] Xu Q., Zheng F., Cao X., Yang P., Xing Y., Zhang P., Liu H., Zhou G., Liu X., Bi X. (2021). Effects of Airflow Ultrafine-Grinding on the Physicochemical Characteristics of Tartary Buckwheat Powder. Molecules.

[38] Zhang L., Chen J., Xu F., Han R., Quan M., Wang L. (2022). Effect of *Tremella fuciformis* on dough structure and rheology, noodle flavor, and quality characteristics. LWT.

[39] Tian L., Roos Y.H., Gómez-Mascaraque L.G., Lu X., Miao S. (2023). *Tremella fuciform* Polysaccharides: Extraction, Physicochemical, and Emulsion Properties at Different pHs. Polymers.

[40] Yang R., Ye Y., Liu W., Liang B., He H., Li X., Ji C., Sun C. (2024). Modification of pea dietary fibre by superfine grinding assisted enzymatic modification: Structural, physicochemical, and functional properties. Int. J. Biol. Macromol..

[41] Li X., Fan M., Huang Q., Zhao S., Xiong S., Zhang B., Yin T. (2020). Effect of wet-media milling on the physicochemical properties of tapioca starch and their relationship with the texture of myofibrillar protein gel. Food Hydrocoll..

[42] Wang W., Shen M., Jiang L., Song Q., Liu S., Xie J. (2020). Influence of *Mesona blumes* polysaccharide on the gel properties and microstructure of acid-induced soy protein isolate gels. Food Chem..

[43] Xing H., Liu X., Hu Y., Hu K., Chen J. (2024). Effect of *Lycium barbarum* polysaccharides on heat-induced gelation of soy protein isolate. Food Hydrocoll..

[44] Cao H., Fan D., Jiao X., Huang J., Zhao J., Yan B., Zhou W., Zhang W., Zhang H. (2018). Effects of microwave combined with conduction heating on surimi quality and morphology. J. Food Eng..

[45] Liu R., Zhao S.-M., Xiong S.-B., Xie B.-J., Qin L.-H. (2008). Role of secondary structures in the gelation of porcine myosin at different pH values. Meat Sci..

[46] Zhuang X., Han M., Jiang X., Bai Y., Zhou H., Li C., Xu X.-L., Zhou G.-H. (2019). The effects of insoluble dietary fiber on myofibrillar protein gelation: Microstructure and molecular conformations. Food Chem..

[47] Zhao H., Chen J., Hemar Y., Cui B. (2020). Improvement of the rheological and textural properties of calcium sulfate-induced soy protein isolate gels by the incorporation of different polysaccharides. Food Chem..

[48] Feng J., Bai X., Li Y., Kong B., Nuerjiang M., Wu K., Li Z., Xia X. (2023). Improvement on gel properties of myofibrillar protein from chicken patty with potato dietary fiber: Based on the change in myofibrillar protein structure and water state. Int. J. Biol. Macromol..

[49] Cayot P., Schenker F., Houzé G., Sulmont-Rossé C., Colas B. (2008). Creaminess in relation to consistency and particle size in stirred fat-free yogurt. Int. Dairy J..

[50] Gomes E.R., Barroso Dos Anjos Pinto C., Stephani R., Fernandes De Carvalho A., Perrone Í.T. (2023). Effect of adding different types of soluble fibre to high-protein yoghurts on water holding capacity, particle size distribution, apparent viscosity, and microstructure. Int. Dairy J..

[51] Zhuang X., Jiang X., Zhou H., Chen Y., Zhao Y., Yang H., Zhou G. (2020). Insight into the mechanism of physicochemical influence by three polysaccharides on myofibrillar protein gelation. Carbohydr. Polym..

[52] Shi Y., Zang M., Wang S., Zhao B., Xu C., Bai J., Zhao Y., Qiao X., Wu J. (2022). Effects of citrus fibre and soybean protein isolate on heat-induced pork myofibrillar protein gel properties under low-sodium salt conditions. Int. J. Food Sci. Technol..

[53] Lu W., Wu D., Wang L., Song G., Chi R., Ma J., Li Z., Wang L., Sun W. (2023). Insoluble dietary fibers from Lentinus edodes stipes improve the gel properties of pork myofibrillar protein: A water distribution, microstructure and intermolecular interactions study. Food Chem..

[54] Zhuang X., Han M., Bai Y., Liu Y., Xing L., Xu X.-L., Zhou G.-H. (2018). Insight into the mechanism of myofibrillar protein gel improved by insoluble dietary fiber. Food Hydrocoll..

[55] Varnaitė L., Keršienė M., Šipailienė A., Kazernavičiūtė R., Venskutonis P.R., Leskauskaitė D. (2022). Fiber-Rich Cranberry Pomace as Food Ingredient with Functional Activity for Yogurt Production. Foods.

[56] Bai X., He Y., Quan B., Xia T., Zhang X., Wang Y., Zheng Y., Wang M. (2022). Physicochemical properties, structure, and ameliorative effects of insoluble dietary fiber from tea on slow transit constipation. Food Chem. X.

[57] Zhu N., Zang M., Wang S., Zhang S., Zhao B., Liu M., Li S., Wu Q., Liu B., Zhao Y. (2022). Modulating the structure of lamb myofibrillar protein gel influenced by psyllium husk powder at different NaCl concentrations: Effect of intermolecular interactions. Food Chem..

[58] Wu X., Zhang Z., Zhang Z., Liu H., Liu G., Yu B., Zhao H., Tao H., Cui B. (2025). Water-insoluble citrus fiber enhances the textural properties and water-holding capacity of thermally generated kappa-carrageenan gels via skeletal reinforcement. Food Hydrocoll..

[59] Mary P.R., Mutturi S., Kapoor M. (2022). Non-enzymatically hydrolyzed guar gum and orange peel fibre together stabilize the low-fat, set-type yogurt: A techno-functional study. Food Hydrocoll..

[60] Tian L., Roos Y.H., Miao S. (2024). Interaction and complex formation of sonicated soluble lentil protein and *Tremella fuciformis* polysaccharide. Food Hydrocoll..

[61] Zhang L., Chen J., Xu F., Han R., Quan M. (2022). Effect of *Tremella fuciformis* and Different Hydrocolloids on the Quality Characteristics of Wheat Noodles. Foods.

[62] Qin X., Yang C., Si J., Chen Y., Xie J., Tang J., Dong X., Cheng Y., Hu X., Yu Q. (2023). Fortified yogurt with high-quality dietary fiber prepared from the by-products of grapefruit by superfine grinding combined with fermentation treatment. Food Sci. Technol..

[63] Chen Y., Cai X.-L., Liu L., Zhang T., Qin L.-K., Jia Y.-L. (2025). Preparation and performance characterization of insoluble dietary fiber-alginate-pea protein ternary composite gels. Food Hydrocoll..

[64] Janssen S.W.P.M., Pouvreau L., de Vries R.J. (2024). Commercial plant protein isolates: The effect of insoluble particles on gelation properties. Food Hydrocoll..

[65] Yang Q., Wang Y.-R., Li-Sha Y.-J., Chen H.-Q. (2021). The effects of basil seed gum on the physicochemical and structural properties of arachin gel. Food Hydrocoll..

[66] McCann T.H., Fabre F., Day L. (2011). Microstructure, rheology and storage stability of low-fat yoghurt structured by carrot cell wall particles. Food Res. Int..

[67] Pereira J., Sathuvan M., Lorenzo J.M., Boateng E.F., Brohi S.A., Zhang W. (2021). Insight into the effects of coconut kernel fiber on the functional and microstructural properties of myofibrillar protein gel system. LWT.

