# Supplementary material for: Effects of Tremella fuciformis Powder with Varying Particle Sizes on the Gel Properties of Soy Yogurt
Source: Foods. 2026 Mar 12;15(6):1000. doi: 10.3390/foods15061000 (PMC13025506; doi:10.3390/foods15061000)
Supplement: Supplementary file 1 [file foods-15-01000-s001.zip › foods-4168070-supplementary.pdf]

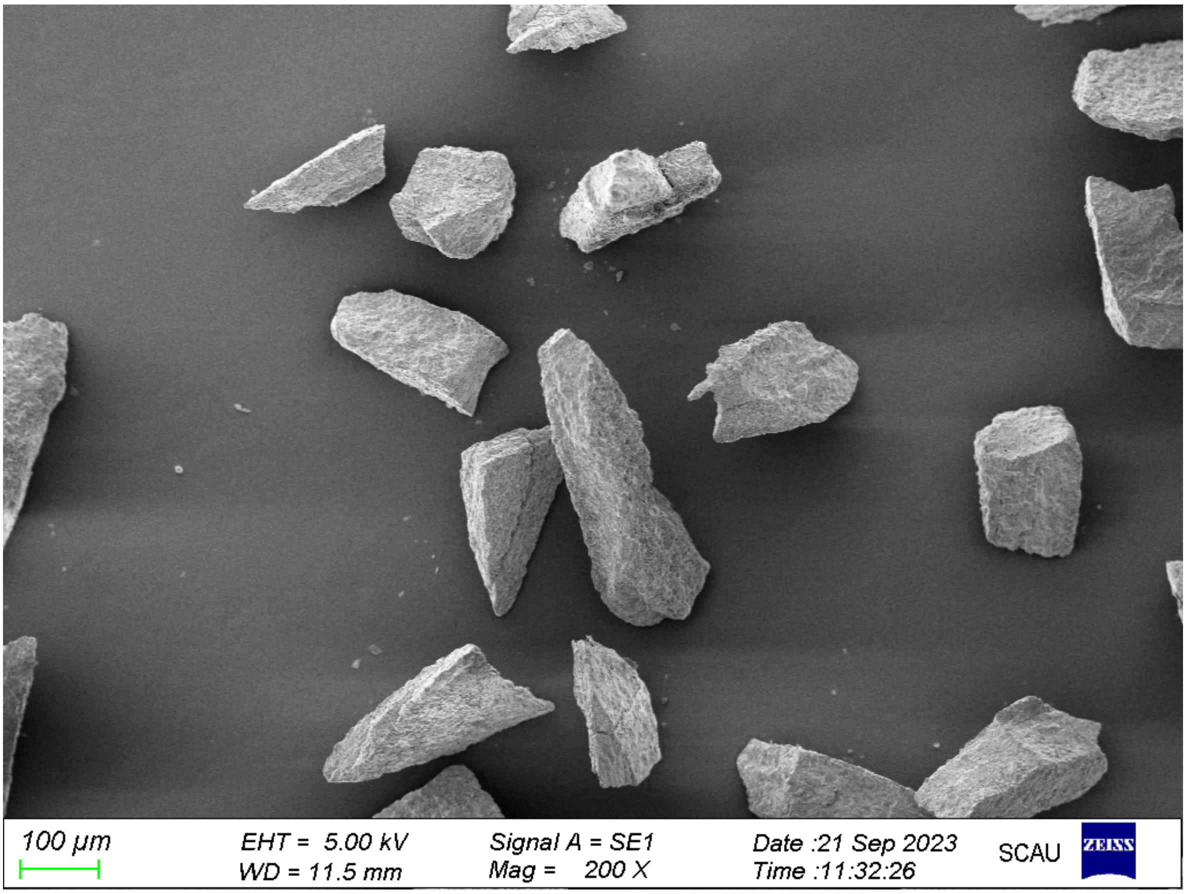

(**a**) Raw SEM images of TFP100 samples

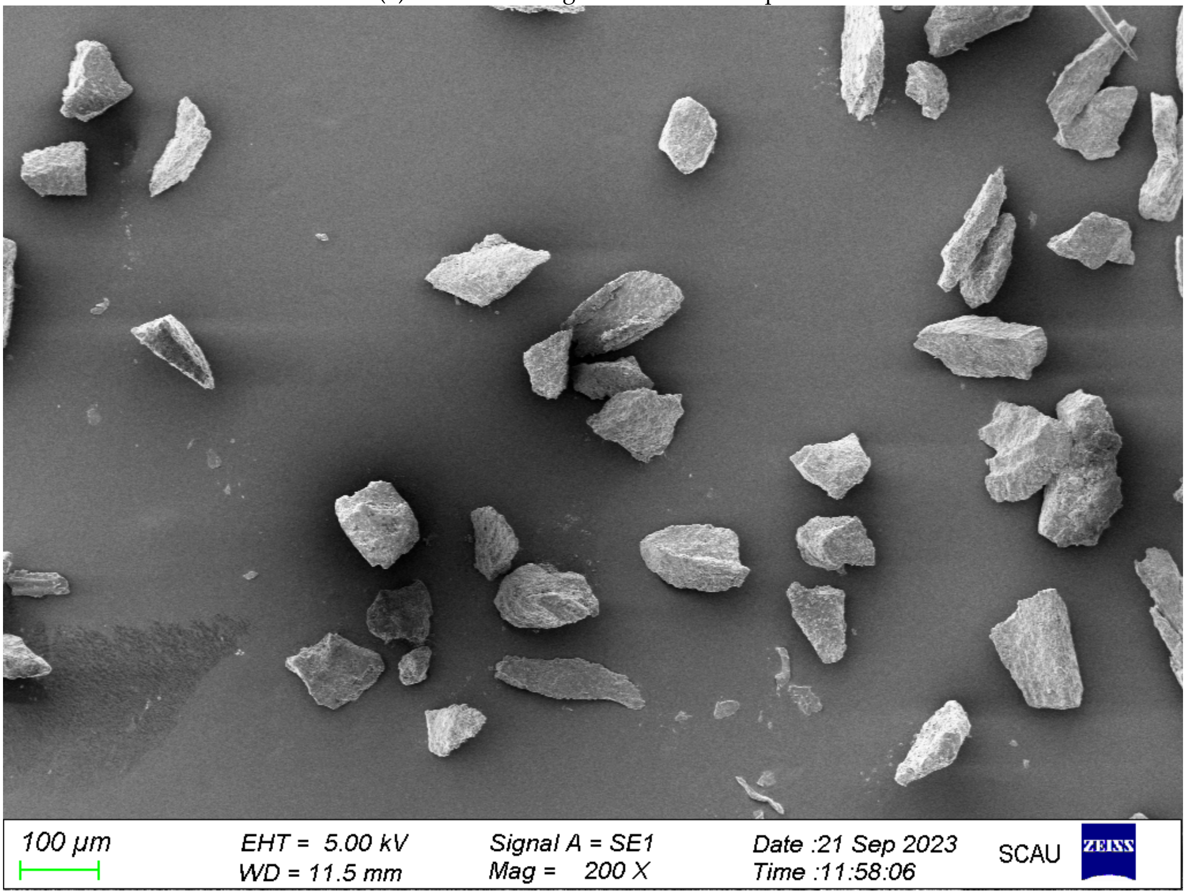

(**b**) Raw SEM images of TFP200 samples

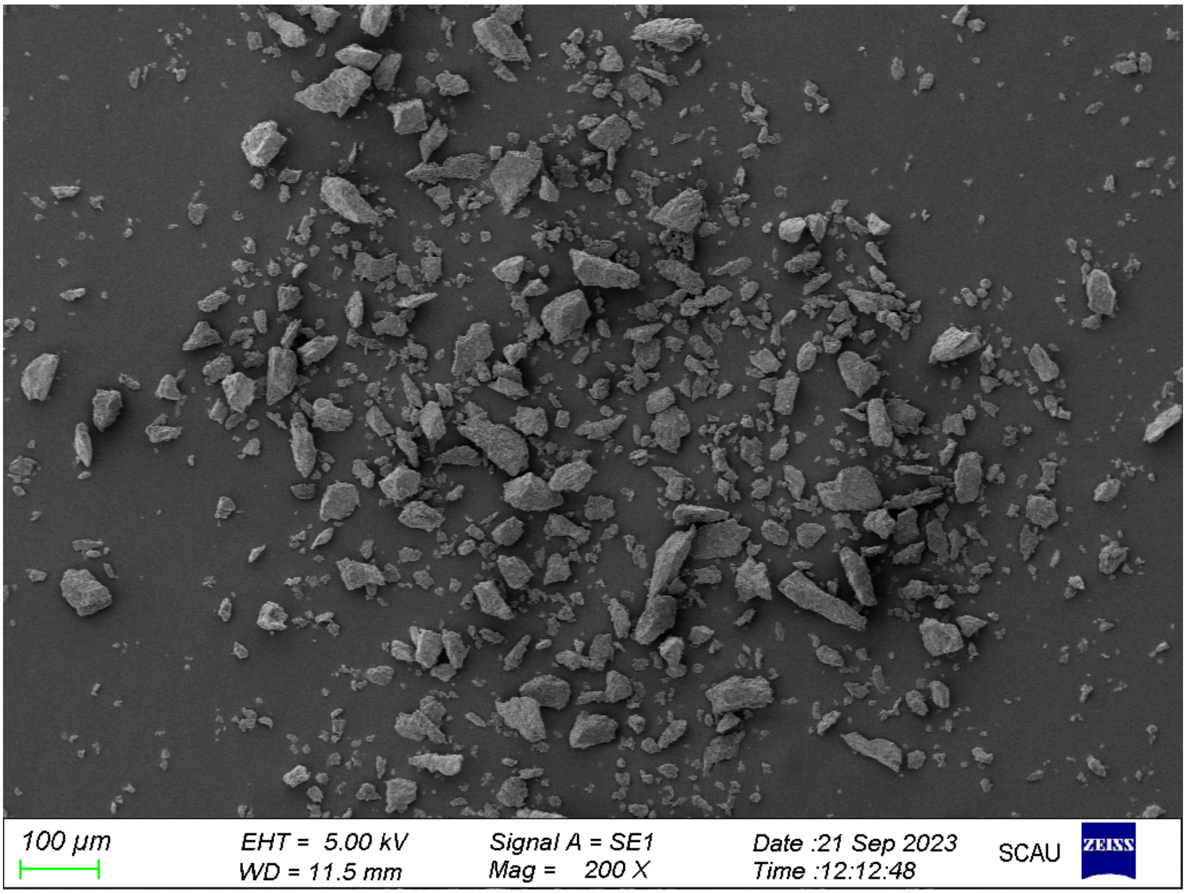

(**c**) Raw SEM images of TFP300 samples

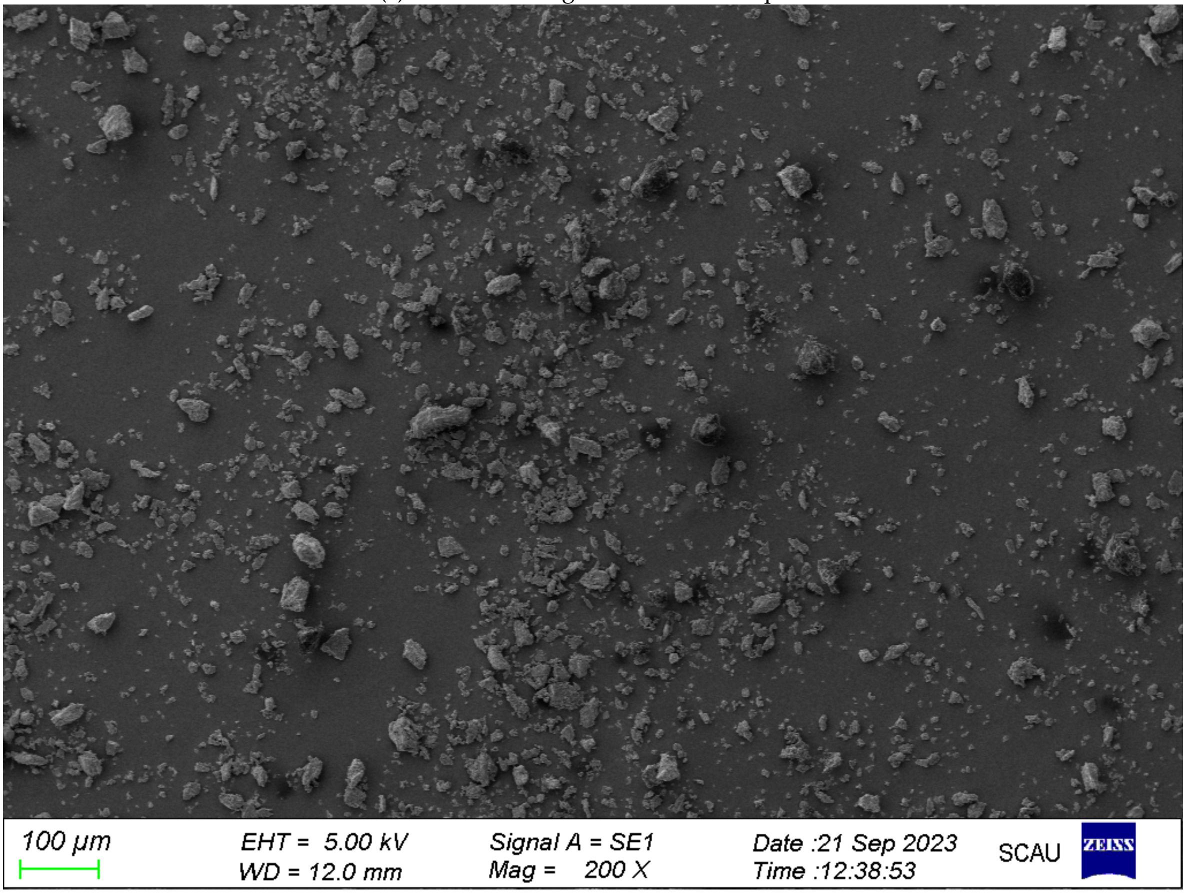

(**d**) Raw SEM images of TFP500 samples

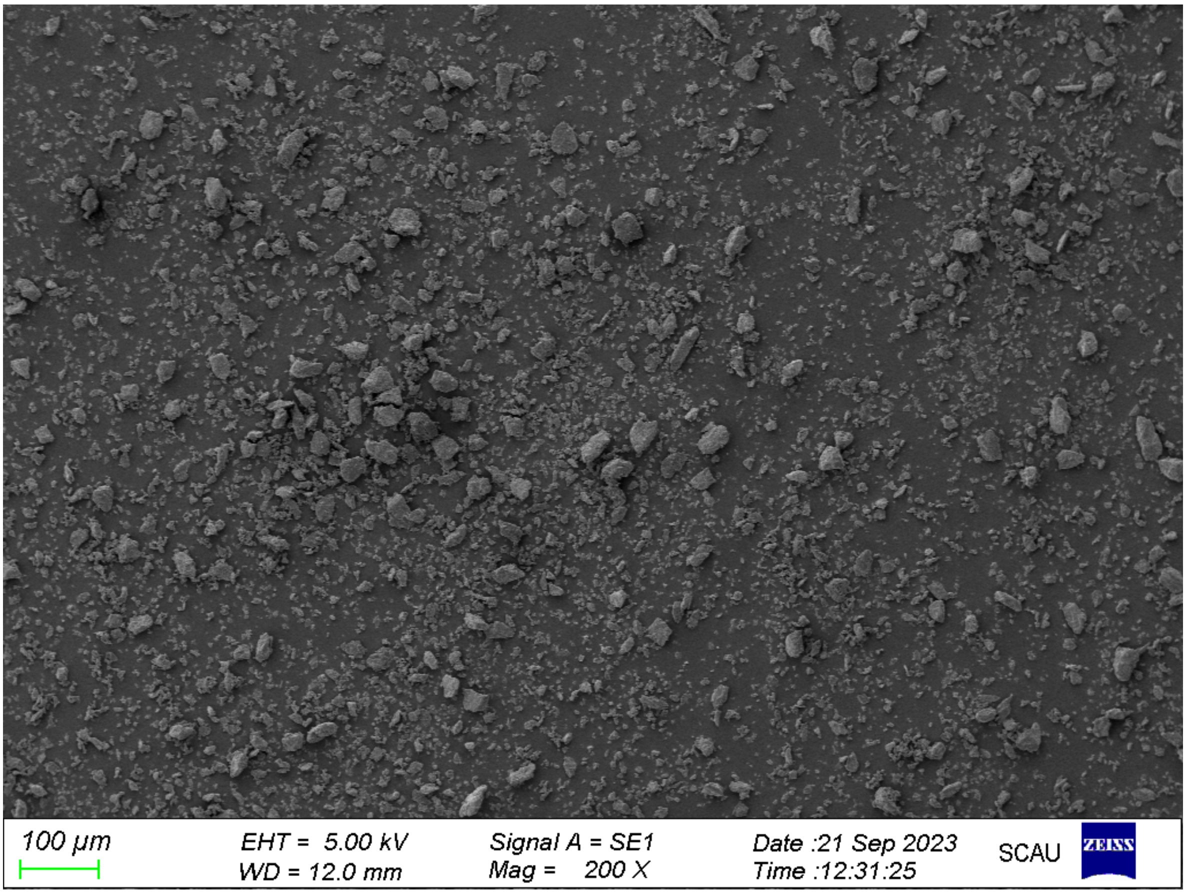

(**e**) Raw SEM images of TFP800 samples

Figure S1: Unprocessed raw SEM images of TFP samples.

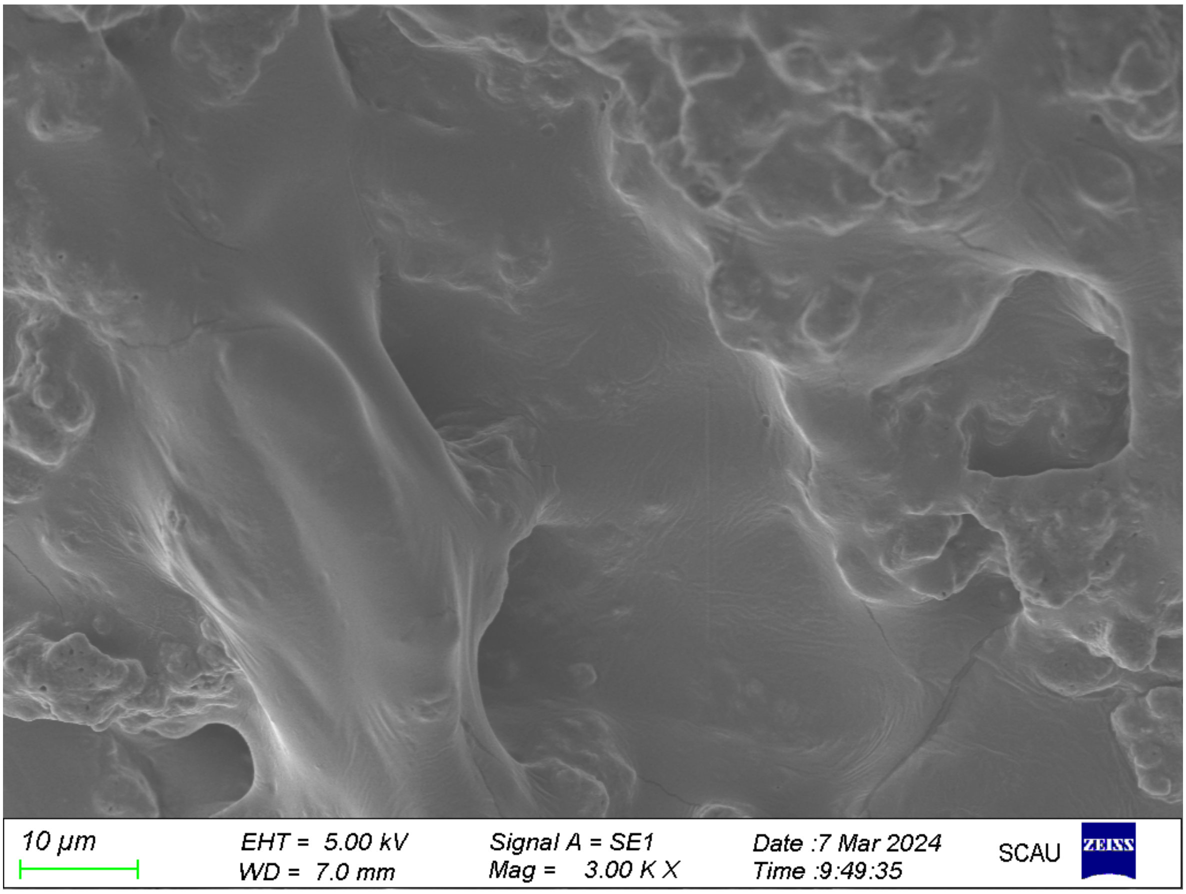

(**a**) raw SEM images of CK sample

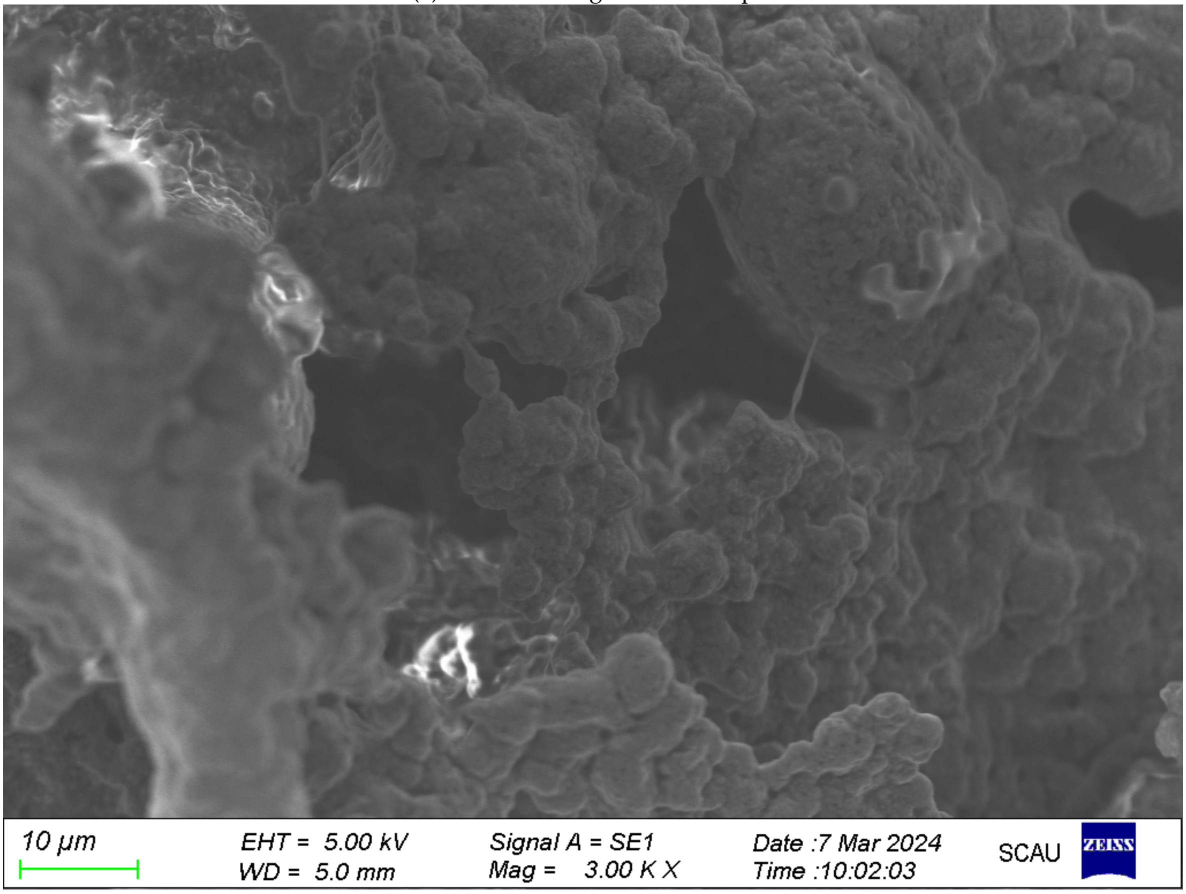

(**b**) raw SEM images of 1%TFP100-SY sample

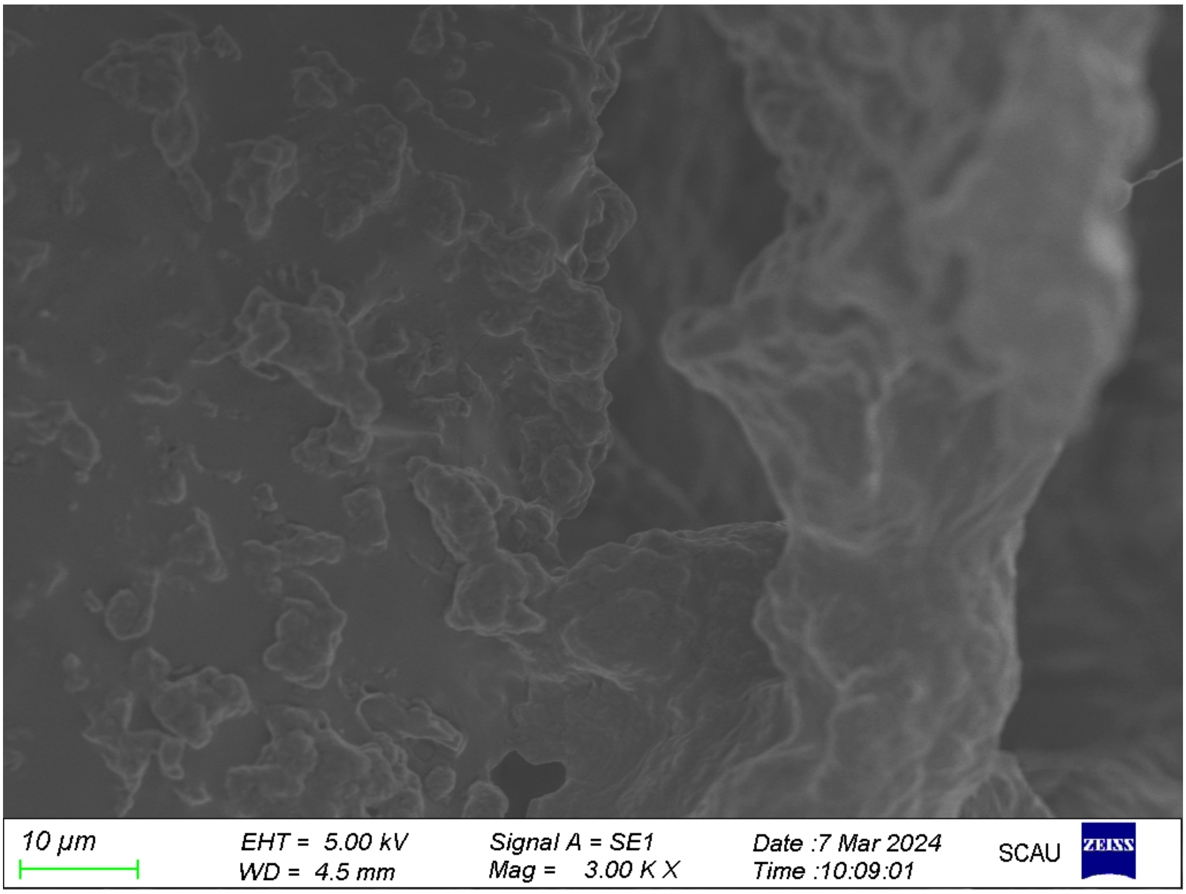

(**c**) raw SEM images of 1.5%TFP100-SY sample

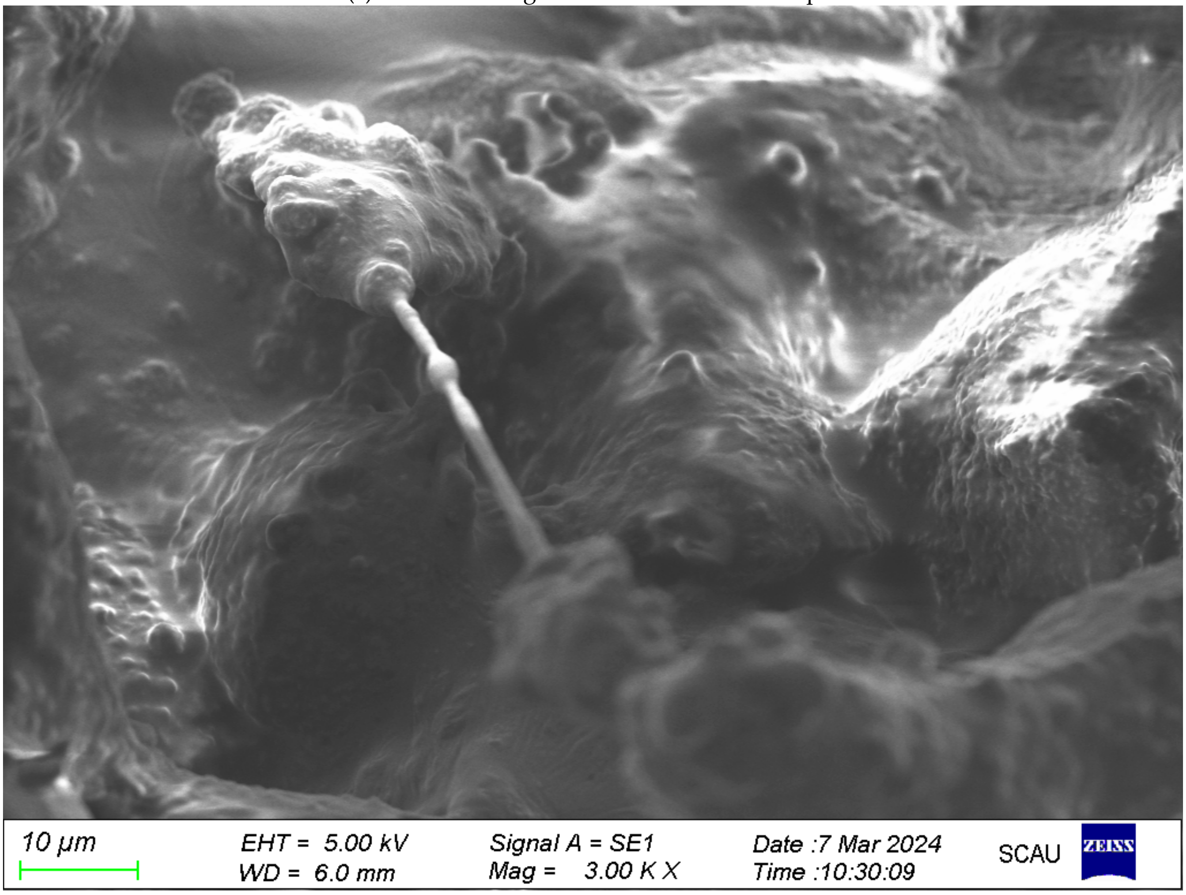

(**d**) raw SEM images of 2%TFP100-SY sample

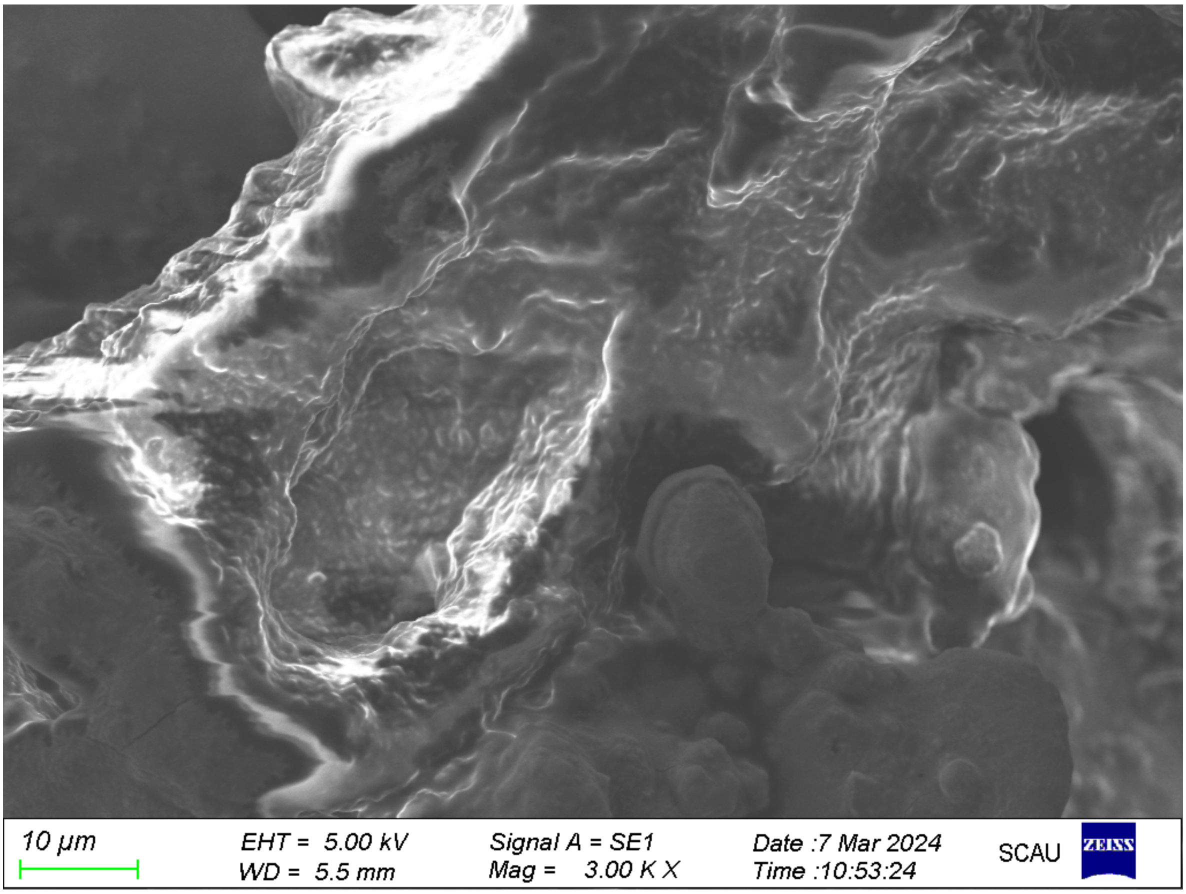

(**e**) raw SEM images of 1%TFP200-SY sample

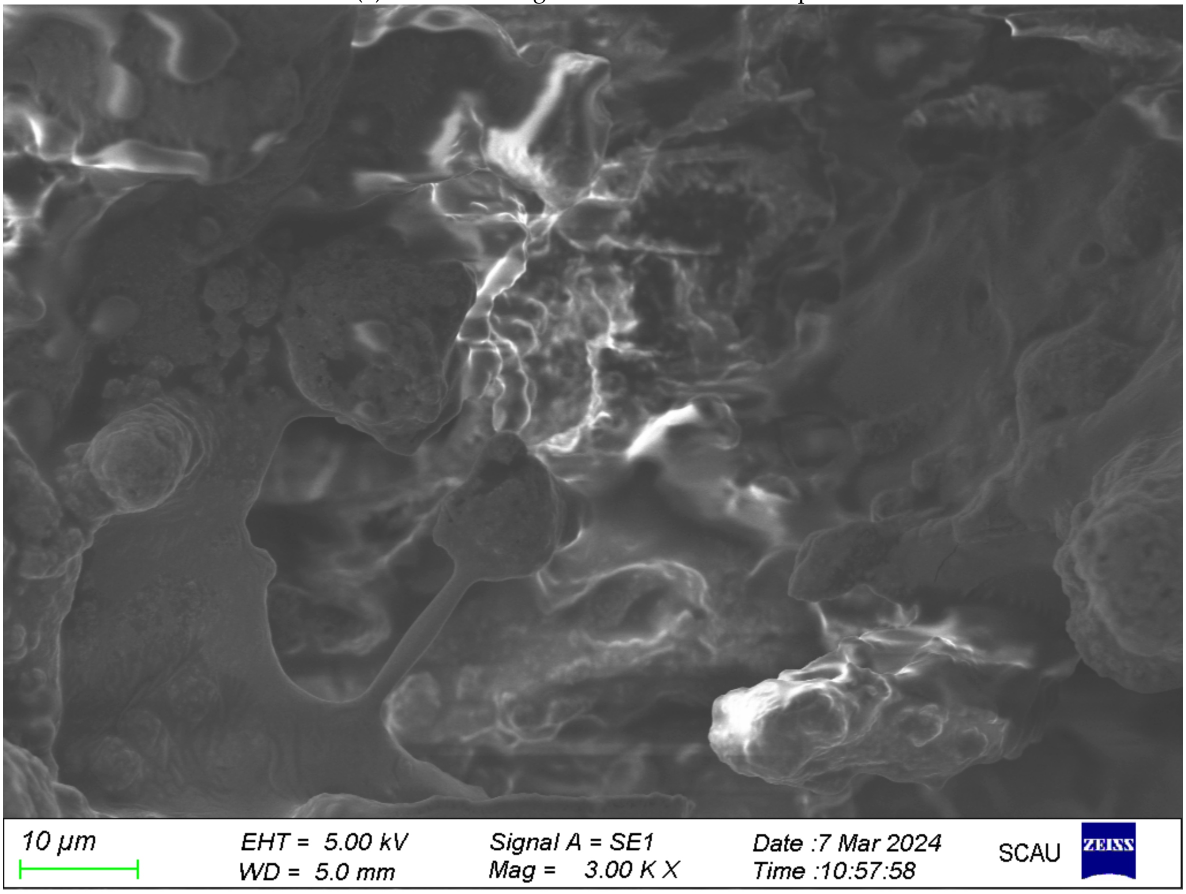

(**f**) raw SEM images of 1.5%TFP200-SY sample

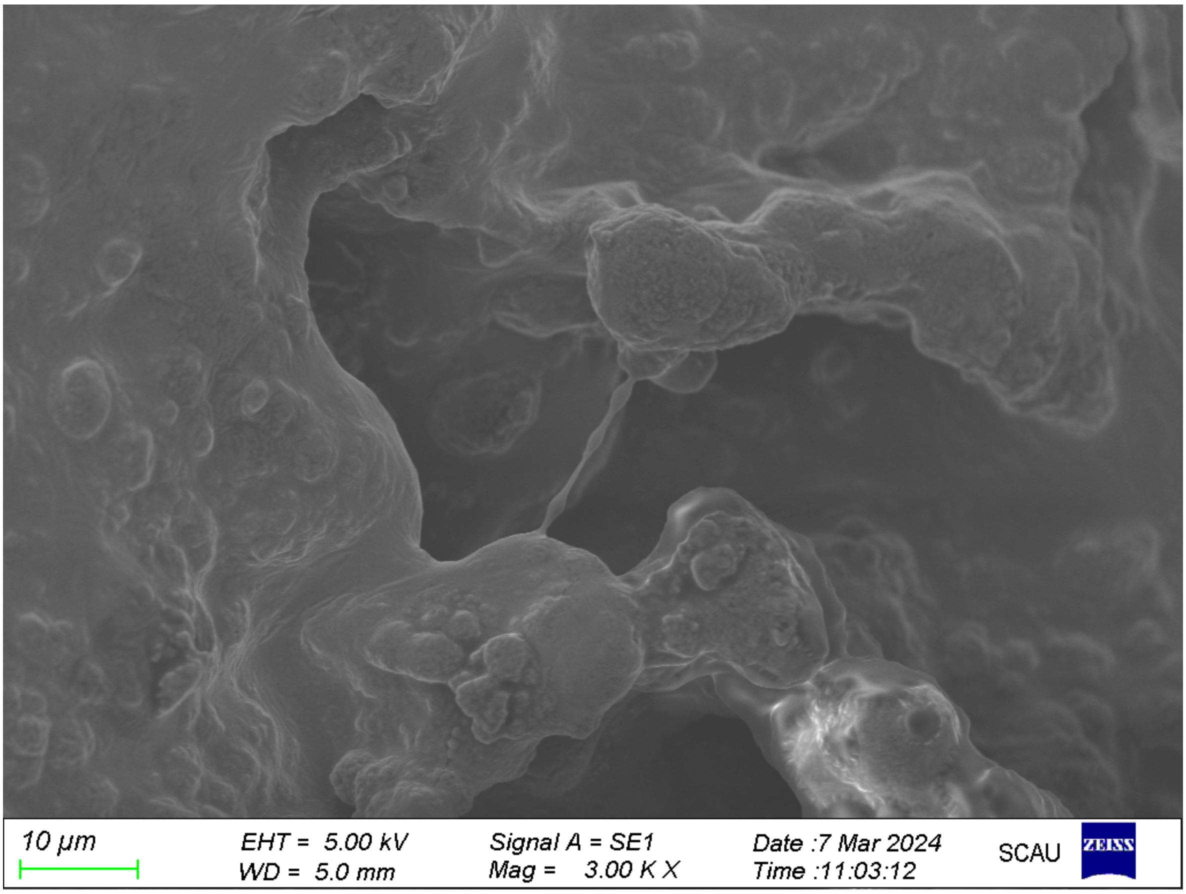

(**g**) raw SEM images of 2%TFP200-SY sample

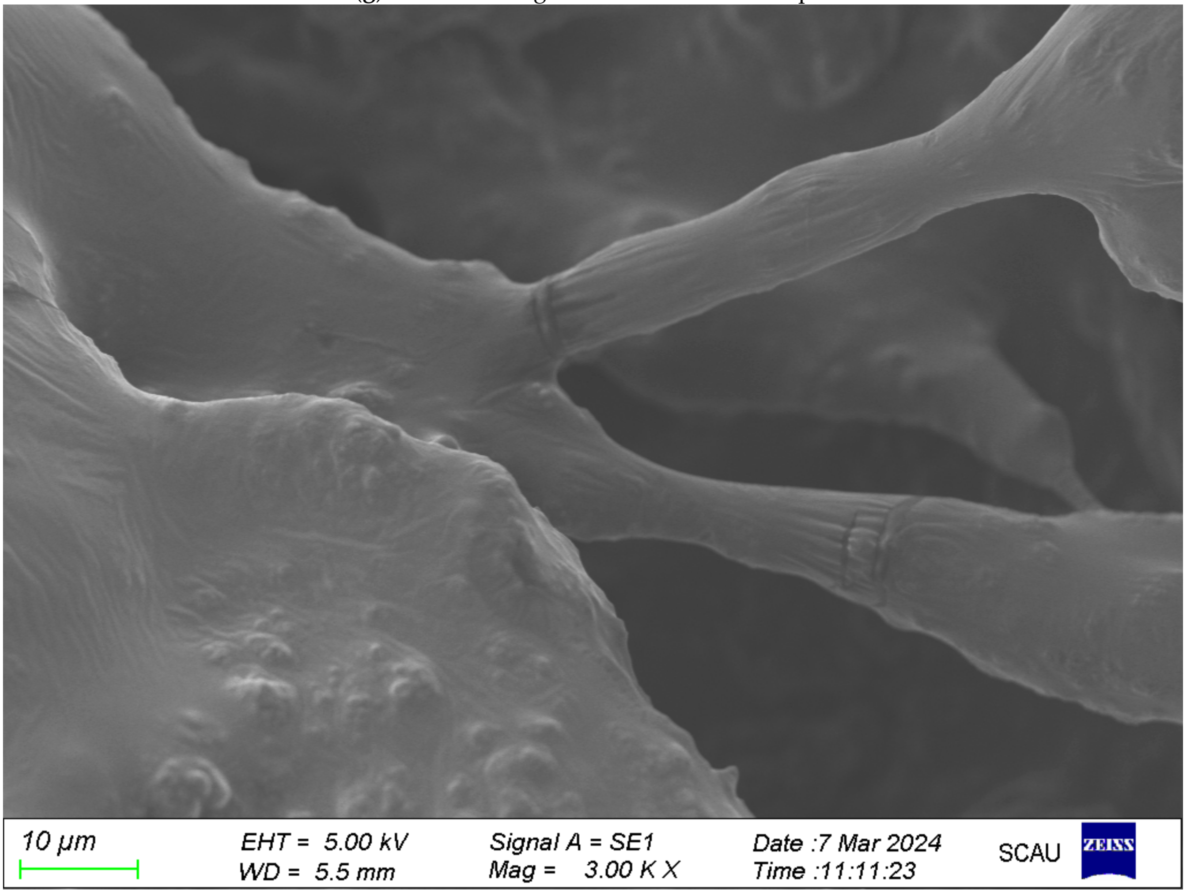

(**h**) raw SEM images of 1%TFP300-SY sample

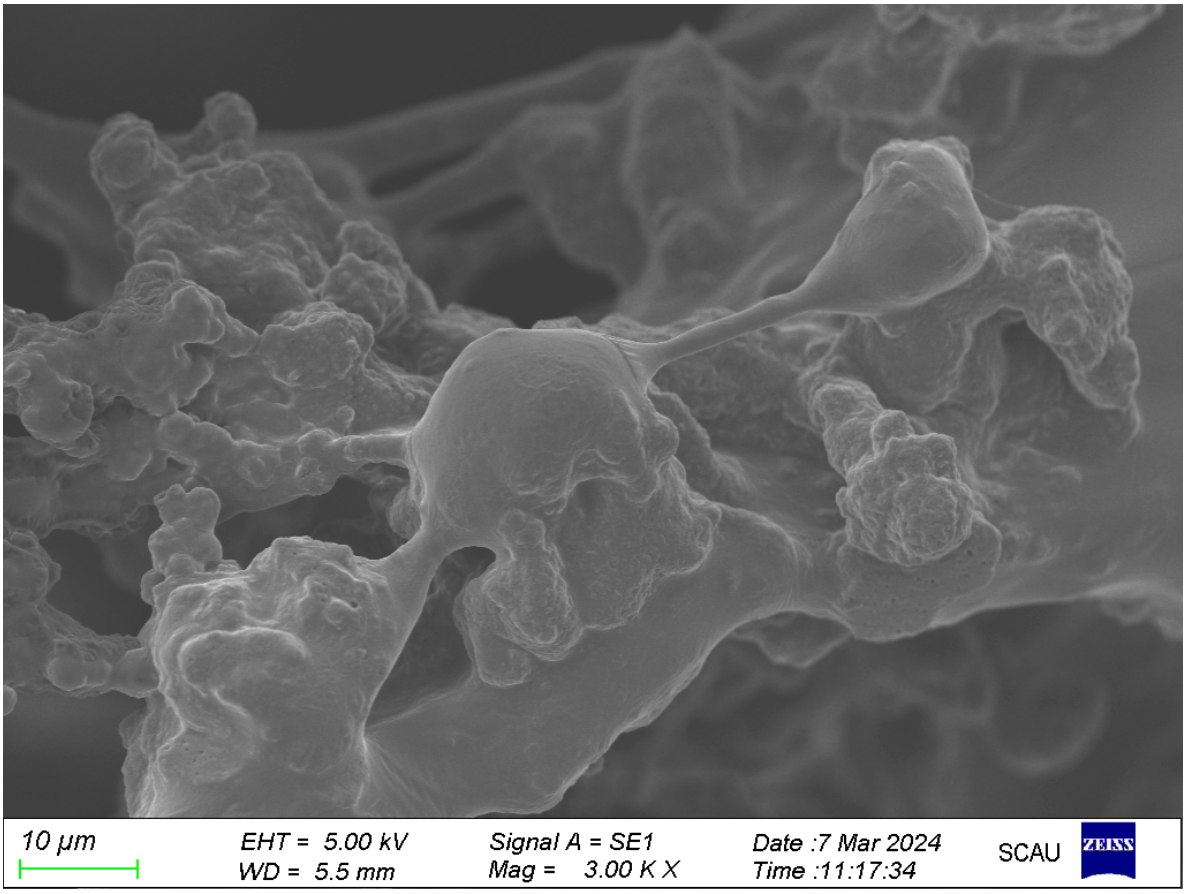

(**i**) raw SEM images of 1.5%TFP300-SY sample

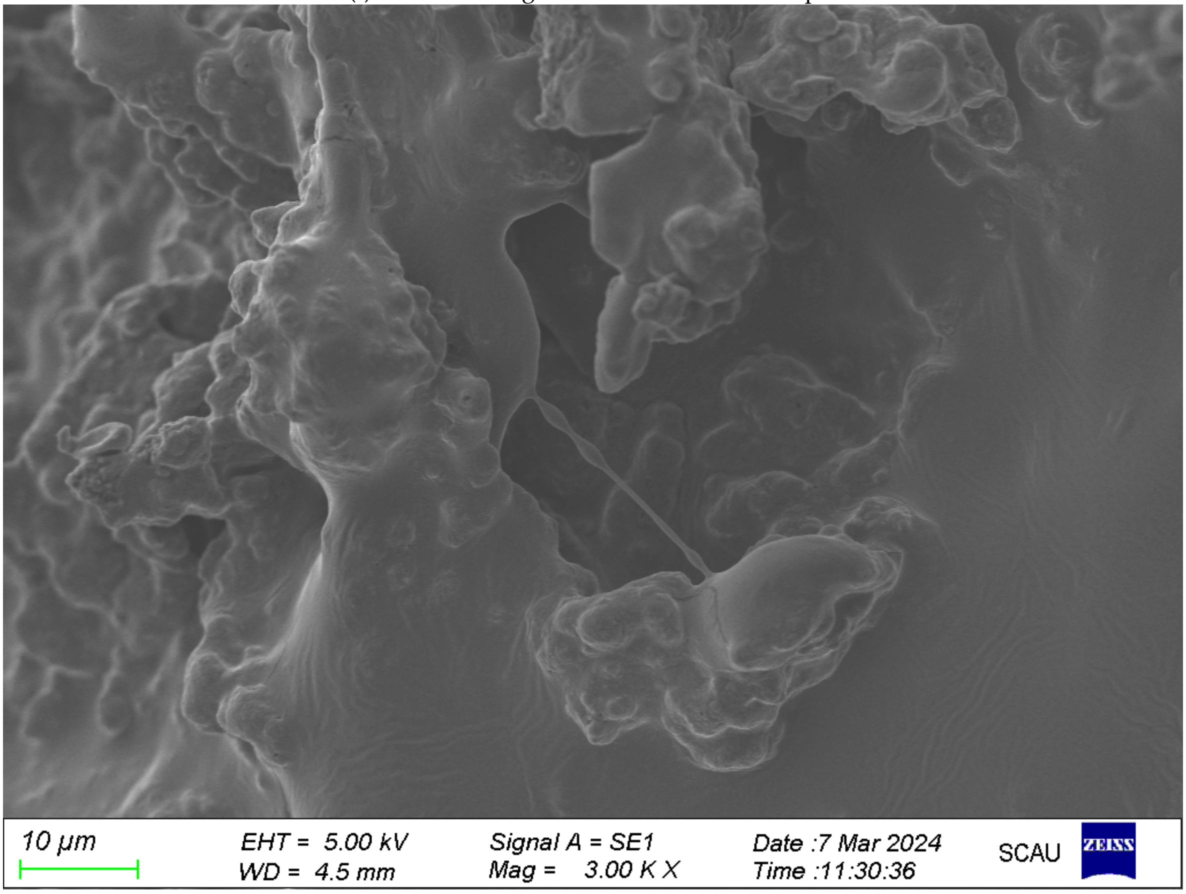

(**j**) raw SEM images of 2%TFP300-SY sample

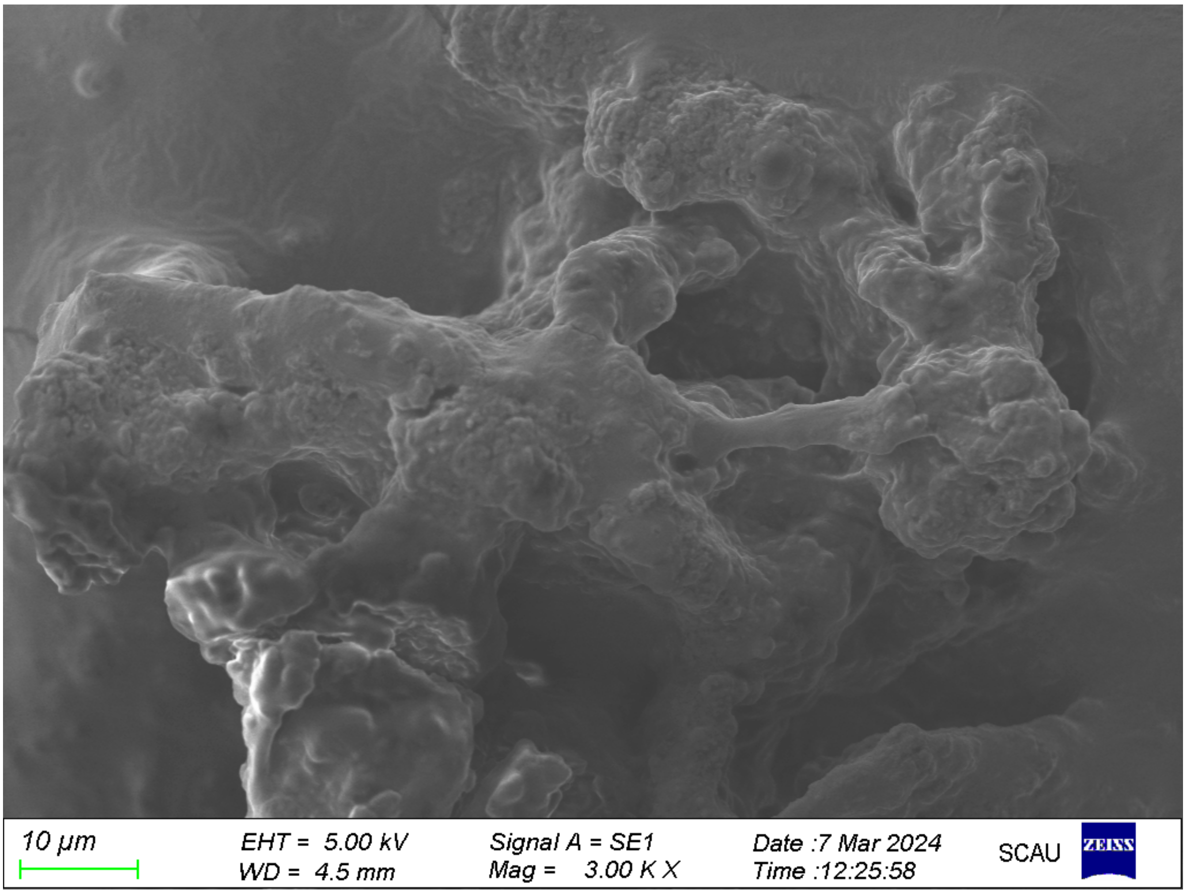

(**k**) raw SEM images of 1%TFP500-SY sample

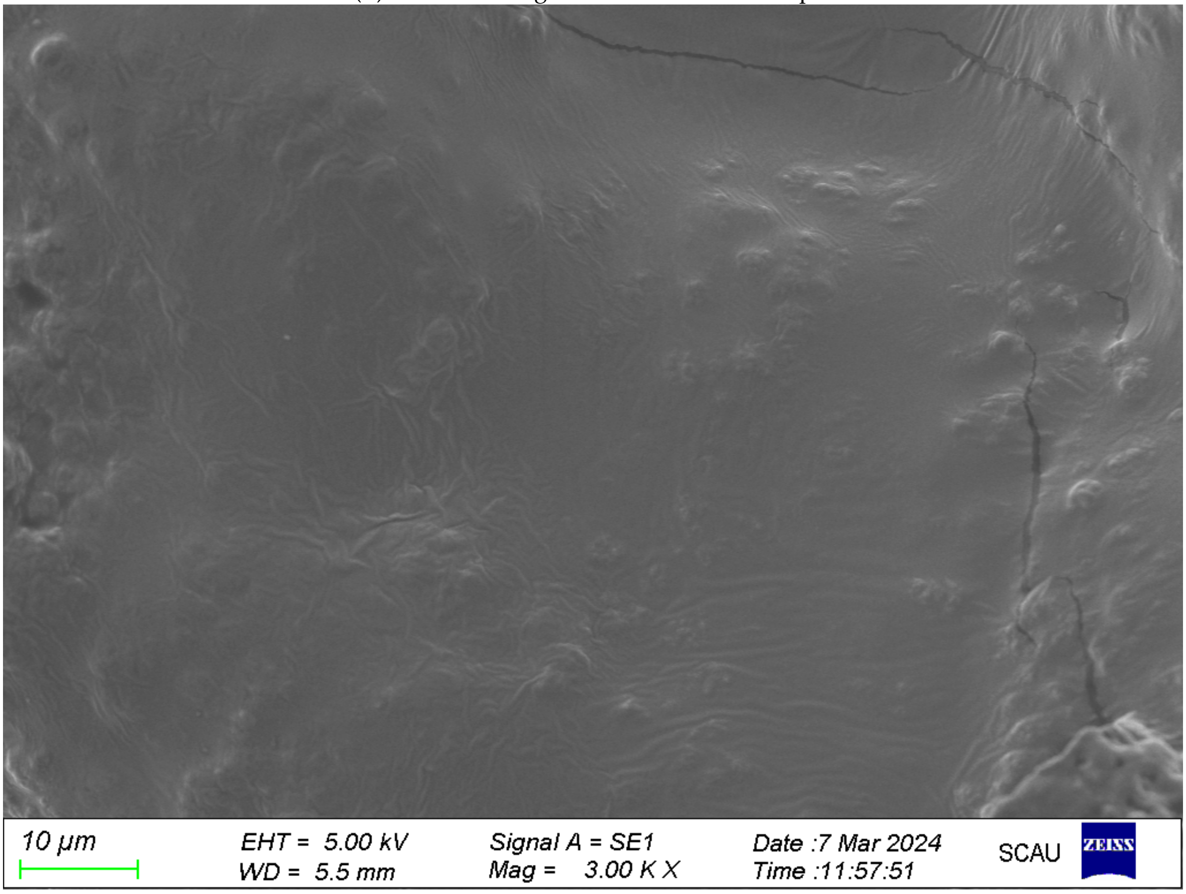

(**l**) raw SEM images of 1.5%TFP500-SY sample

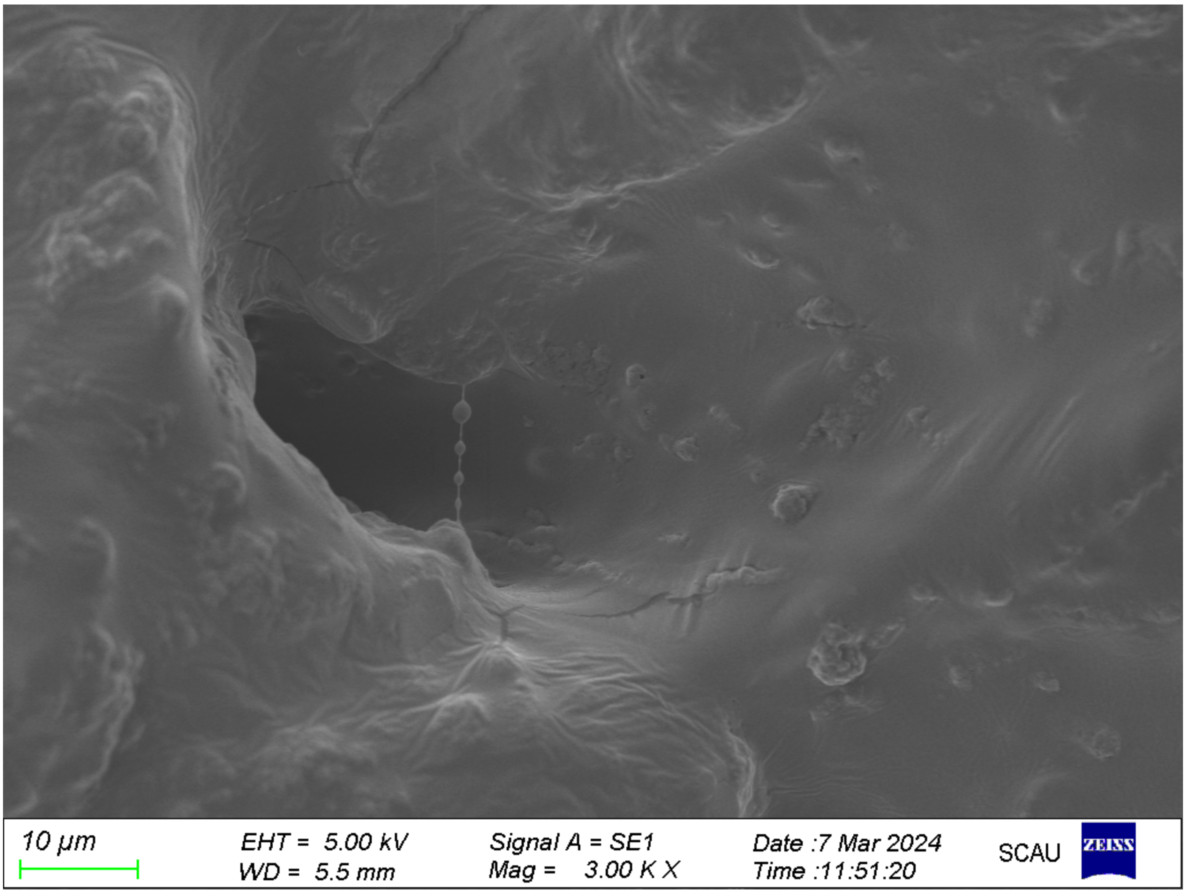

(**m**) raw SEM images of 2%TFP500-SY sample

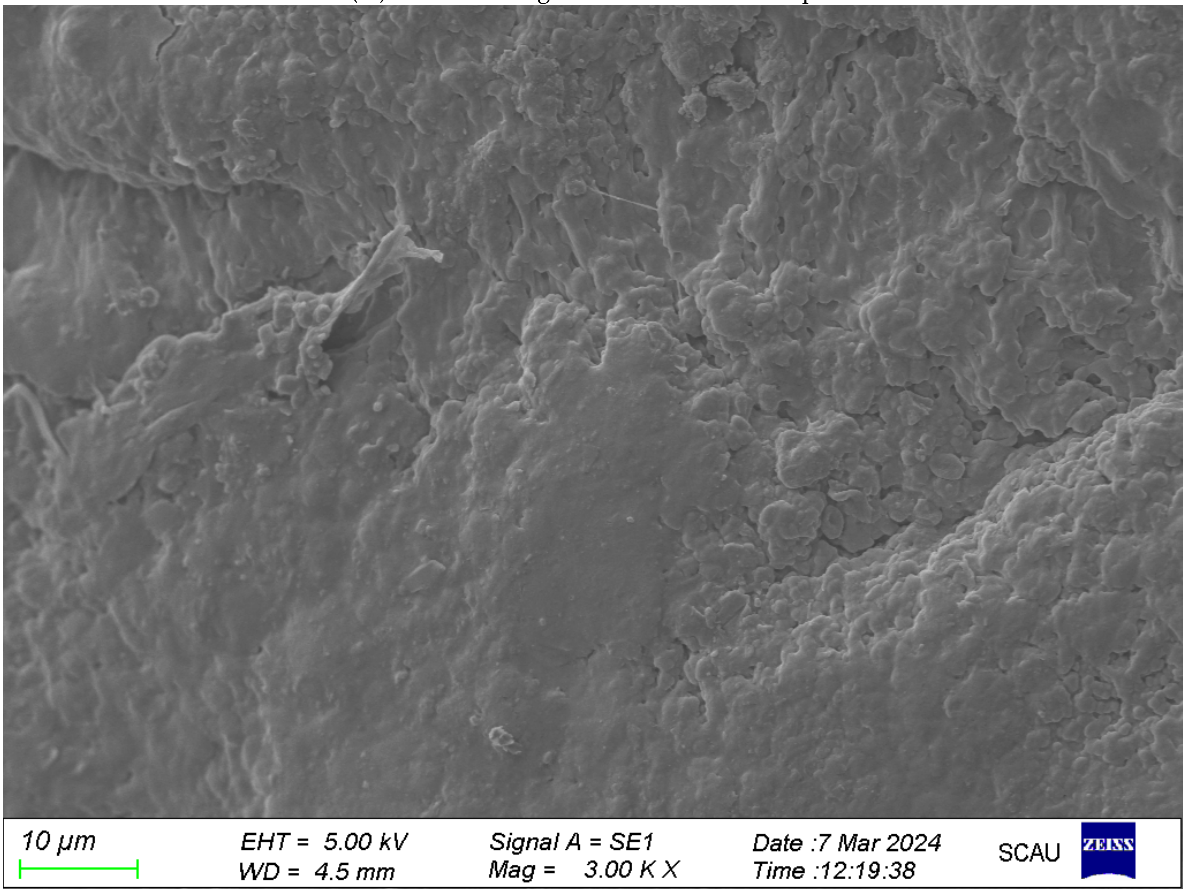

(**n**) raw SEM images of 1%TFP800-SY sample

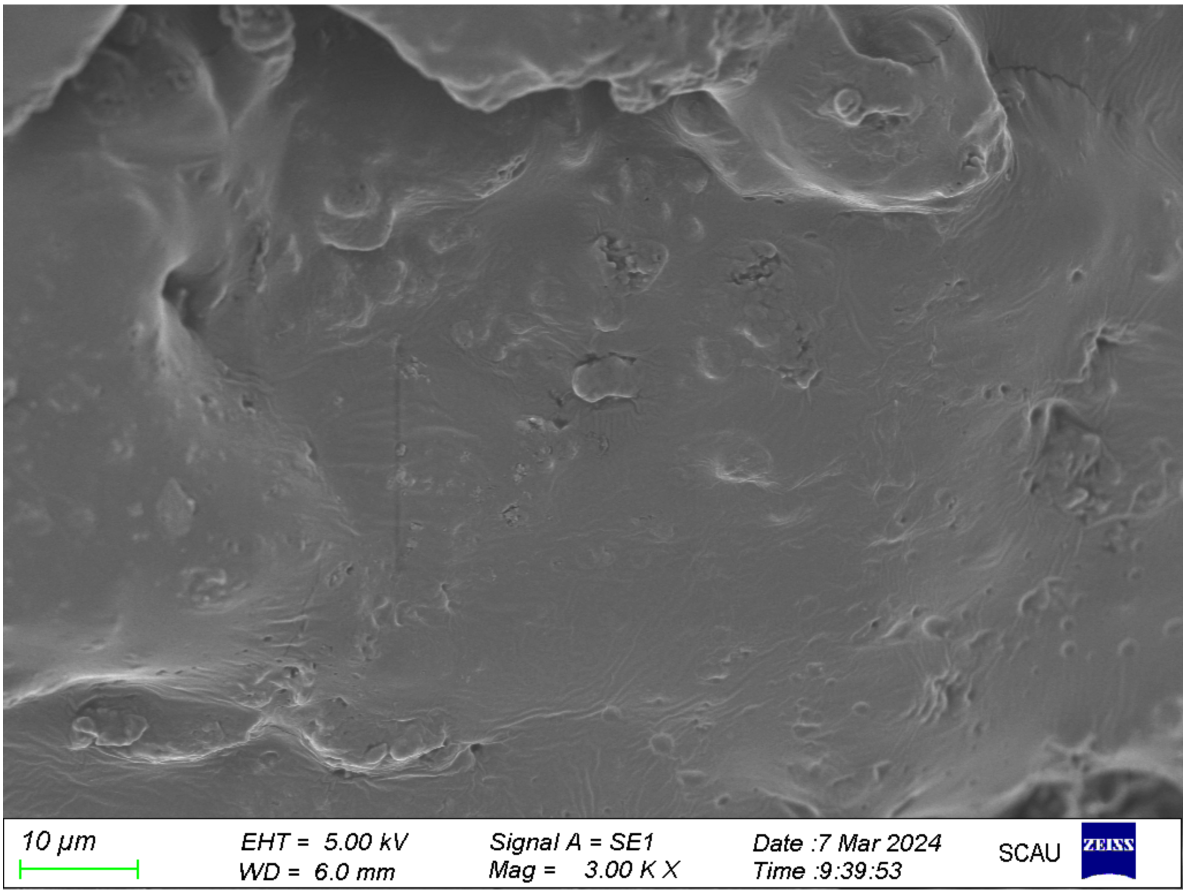

(**o**) raw SEM images of 1.5%TFP800-SY sample

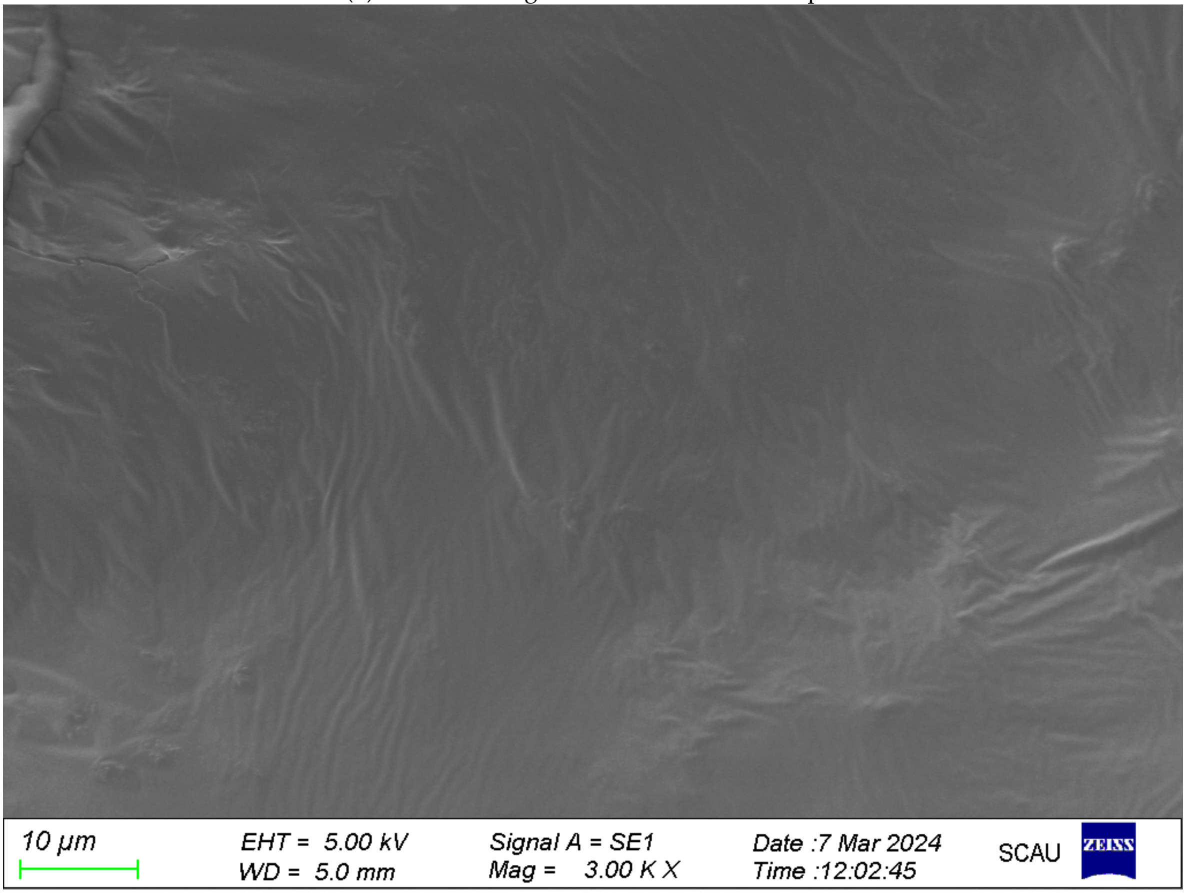

(**p**) raw SEM images of 2%TFP800-SY sample

Figure S2: Unprocessed raw SEM images of soy yogurt samples.
